# An exome sequencing based approach for genome-wide association studies in the dog

**DOI:** 10.1038/s41598-017-15947-9

**Published:** 2017-11-15

**Authors:** Bart J. G. Broeckx, Thomas Derrien, Stéphanie Mottier, Valentin Wucher, Edouard Cadieu, Benoît Hédan, Céline Le Béguec, Nadine Botherel, Kerstin Lindblad-Toh, Jimmy H. Saunders, Dieter Deforce, Catherine André, Luc Peelman, Christophe Hitte

**Affiliations:** 1 Laboratory of Animal Genetics, Faculty of Veterinary Medicine, Ghent University, Merelbeke, Belgium; 2 Institut de Génétique et Développement de Rennes, CNRS-URM6290, Université Rennes1, Rennes, France; 3 Broad Institute of MIT and Harvard, Cambridge, Massachusetts, USA; 4 Science for Life Laboratory, Department of Medical Biochemistry and Microbiology, Uppsala University, Uppsala, Sweden; 5 Department of Medical Imaging and Orthopedics, Faculty of Veterinary Medicine, Ghent University, Merelbeke, Belgium; 6 Laboratory of Pharmaceutical Biotechnology, Faculty of Pharmaceutical Sciences, Ghent University, Ghent, Belgium

## Abstract

Genome-wide association studies (GWAS) are widely used to identify loci associated with phenotypic traits in the domestic dog that has emerged as a model for Mendelian and complex traits. However, a disadvantage of GWAS is that it always requires subsequent fine-mapping or sequencing to pinpoint causal mutations. Here, we performed whole exome sequencing (WES) and canine high-density (cHD) SNP genotyping of 28 dogs from 3 breeds to compare the SNP and linkage disequilibrium characteristics together with the power and mapping precision of exome-guided GWAS (EG-GWAS) versus cHD-based GWAS. Using simulated phenotypes, we showed that EG-GWAS has a higher power than cHD to detect associations within target regions and less power outside target regions, with power being influenced further by sample size and SNP density. We analyzed two real phenotypes (hair length and furnishing), that are fixed in certain breeds to characterize mapping precision of the known causal mutations. EG-GWAS identified the associated exonic and 3′UTR variants within the *FGF5* and *RSPO2* genes, respectively, with only a few samples per breed. In conclusion, we demonstrated that EG-GWAS can identify loci associated with Mendelian phenotypes both within and across breeds.

## Introduction

Aside from its special place as a companion animal, the dog or *Canis lupus familiaris* is also genetically exceptional. While population bottlenecks, the popular sire effect and stringent breeding programs have led to an extreme phenotypic diversity, they resulted at the same time in a high number of genetic disorders^1,2^. Several disorders are breed-specific, others are more widely dispersed through the population. A large number of these disorders share clinical and laboratory abnormalities with human diseases and over 50% of the genetic diseases in the dog are considered to be potential models for human diseases according to the Online Mendelian Inheritance in Animals database^3^.

To identify the causal mutations responsible for these traits, two different approaches are often used. In genome-wide association studies (GWAS), it is generally not the causal mutation that is directly genotyped. Instead, tag Single Nucleotide Polymorphisms (tagSNPs) are genotyped. Definition wise, any variant that is in linkage disequilibrium (LD) with other variants in the neighborhood and that as such can be used to tag the mutational variability in that region, can be seen as a tagSNP. Because of the LD between a tagSNP and the causal SNP, associations between a phenotype and the causal SNP can be found, even though the causal variant itself is not genotyped. When GWAS is used that way, it is an indirect method and requires further steps downstream (e.g. fine mapping, candidate gene sequencing) to detect the causal mutation. This is in contrast to whole exome sequencing (WES) and whole genome sequencing (WGS) where one tries to identify the disease-causing mutation directly, often by the sequential use of a set of heuristic filters^4^. Aside from the method used, they also differ in price and initial hypothesis: while GWAS and WGS are supposed to give a general overview of the genome, WES focuses on pre-specified target regions, generally the exonic part of protein-coding regions (even though non-coding RNAs are also included in some WES designs)^5^.

Together with huge phenotypical consequences, the population history of dogs has also left its marks at the level of the genome: within dog breeds, LD is often extensive up to 2 Mb and far higher compared to the human genome^2,6,7^. Taking advantage of this long-range LD, mapping of disease loci with GWAS becomes highly efficient, even with a relatively sparse number of tagSNPs^8^. As the LD is far less between breeds, fine mapping can next be achieved by combining different breeds^8^. Also for WES, the high degree of relatedness of dogs within breeds is beneficiary: with a limited sample size, variant reduction is substantial and should allow pinpointing the causal allele quickly^9^.

With the recent introduction of WES in the dog, several modern tools are currently available to identify disease-causing mutations^10^. Whereas WES is generally considered a direct strategy to identify causal variants, recently, an LD-based WES approach has been proposed^11^. In this approach, associations are detected between phenotypes and causal variants by exploiting the LD between this causal variant and WES variants discovered during sequencing. Here, WES variants are also used to tag surrounding causal variants, i.e. they can be seen as tagSNPs. In this paper, we investigate in detail the mapping power and precision of this LD-based WES approach within the Poodle breed and for fixed traits across multiple dog breeds.

To enable a direct comparison, 28 samples from three different breeds (16 Poodles, 6 Labrador retrievers and 6 Golden retrievers) underwent genotyping (Illumina canine high-density (cHD) 170k Whole-Genome Genotyping BeadChip) and were also sequenced with the exome-1.0, the first canine WES design published^10,12^. The tagSNP characteristics were assessed and the LD was compared. Next, power analyses based on simulated phenotypes were conducted to analyze the influence of sample size, SNP density and causal variant location within the Poodle breed. The power of the association was also investigated for long intergenic non-coding RNAs (lincRNAs), which are regulatory regions residing outside the exome that have for example been implicated in cancer^13,14^. Finally, using real phenotypes, the mapping precision of EG-GWAS to identify variants inside the *RSPO2* and *FGF5* genes, associated with two coat types in dogs was investigated^15,16^. As these coat phenotypes are fixed within breeds, they demonstrate the power and precision of WES for mapping across multiple breeds.

## Results

### SNP characteristics and genome-wide LD comparison of cHD and WES data

**Figure 1:**
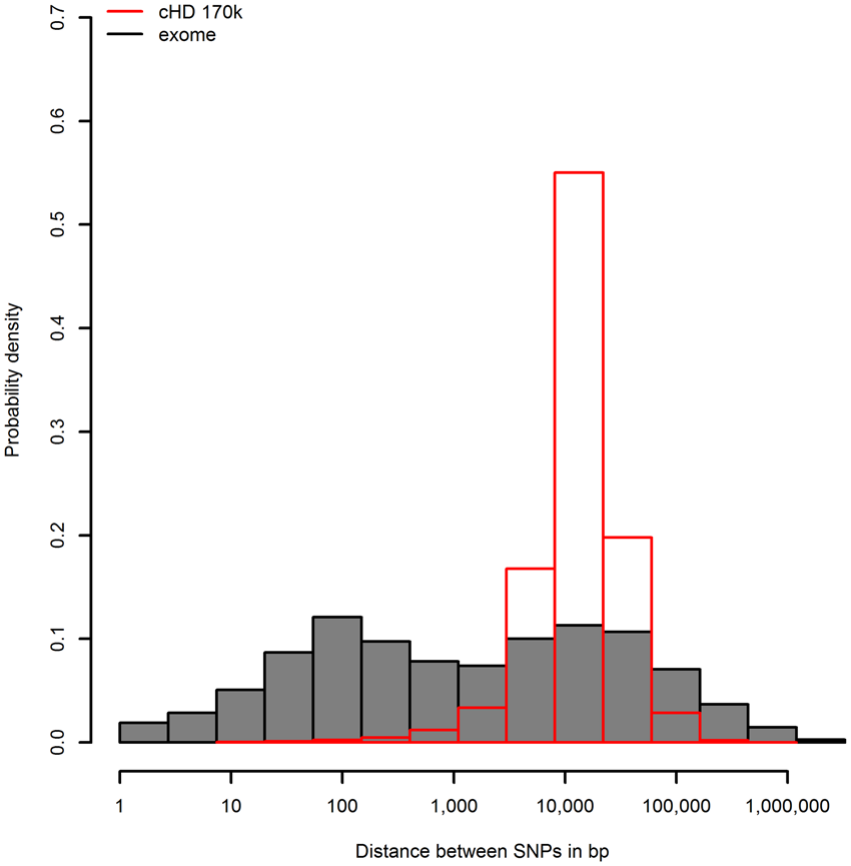
Distribution of distance between subsequent SNPs for the exome-1.0 and canine high-density array (chromosome 1). Only those SNPs that passed the filters for linkage disequilibrium calculations were used (sufficiently polymorphic, sufficient call rate (see methods section)). Distances are expressed in bp.

**Figure 2:**
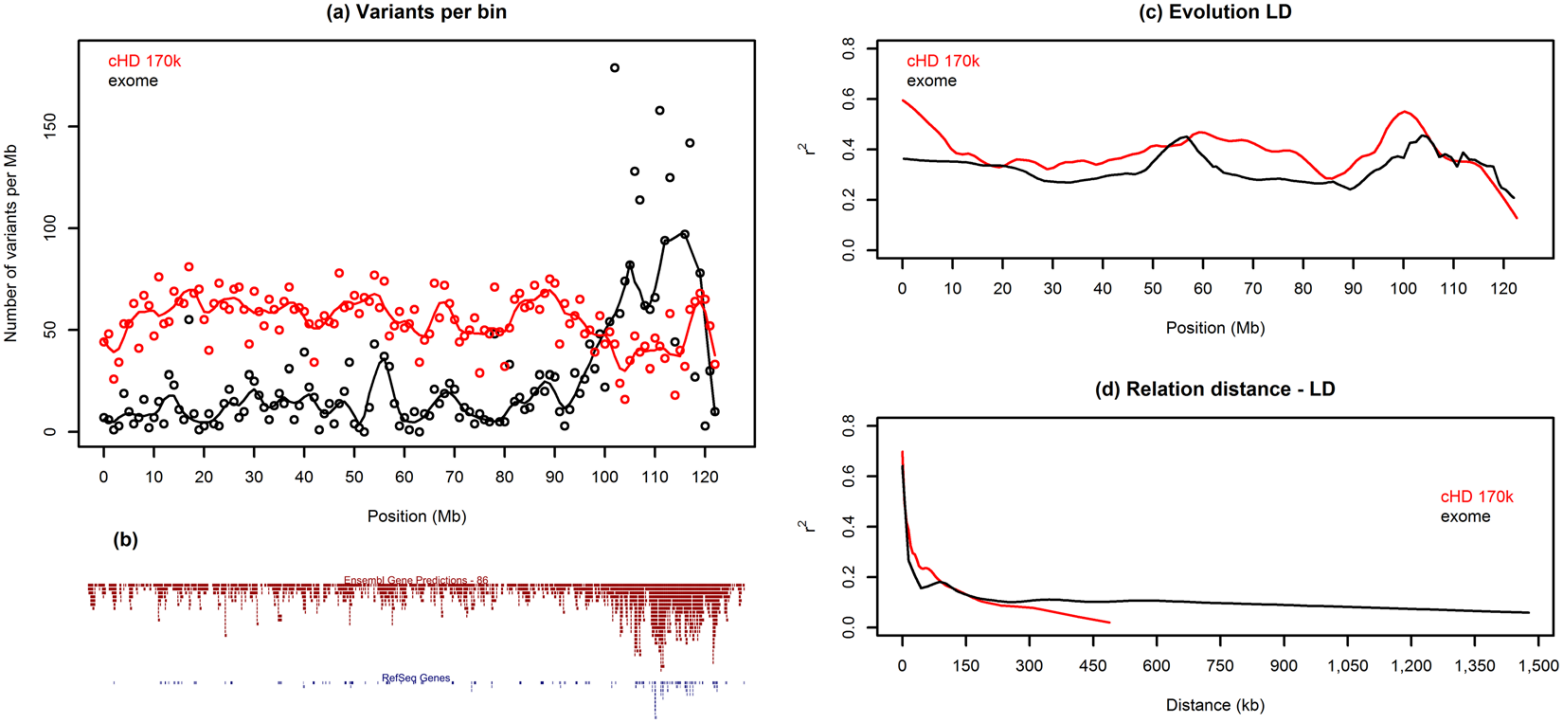
Relation between linkage disequilibrium, gene annotation, SNP density and distance between tagSNPs on chromosome 1. (**a**) Whole exome sequencing (WES)- and canine high-density array (cHD)-specific informative SNP count per bin (binsize: 1 Mb) relative to position. (**b**) Overview of RefSeq Genes track (blue) and Ensembl Gene Predictions track (brown) density relative to position. (**c**) WES- and cHD-specific linkage disequilibrium (measured in r²) relative to position. (**d**) Relation between r² and distance between subsequent SNPs. In each graph, lines are obtained with LOWESS (locally weighed scatterplot smoothing).

A direct comparison of the physical distance of cHD tagSNPs and WES informative SNPs that passed the filters for LD calculation, shows that whereas the mean distance between WES SNPs (1 SNP every 40 kb) is larger relative to cHD SNPs (1 SNP every 17 kb), the median distance is smaller (WES: 1 SNP every 1 kb, cHD: 1 SNP every 13 kb), indicating a highly skewed distance distribution (Fig. 1). To analyze the variability of the SNP density, all chromosomes were binned in non-overlapping regions of 1 Mb. The number of variants per bin varies far more for WES relative to the more regular cHD SNP density, as detailed in Fig. 2(a) for chromosome 1. Even though overall the total number of cHD SNPs surpasses the number of sequence variants for each chromosome (Suppl. Table S1), at certain positions in individual bins, WES has more variants as exemplified towards the end of chromosome 1 (Fig. 2(a)). A comparison with the canfam3.1 annotation reveals an association with gene density, which can be expected given the definition and purpose of WES (Fig. 2(b)).

**Figure 3:**
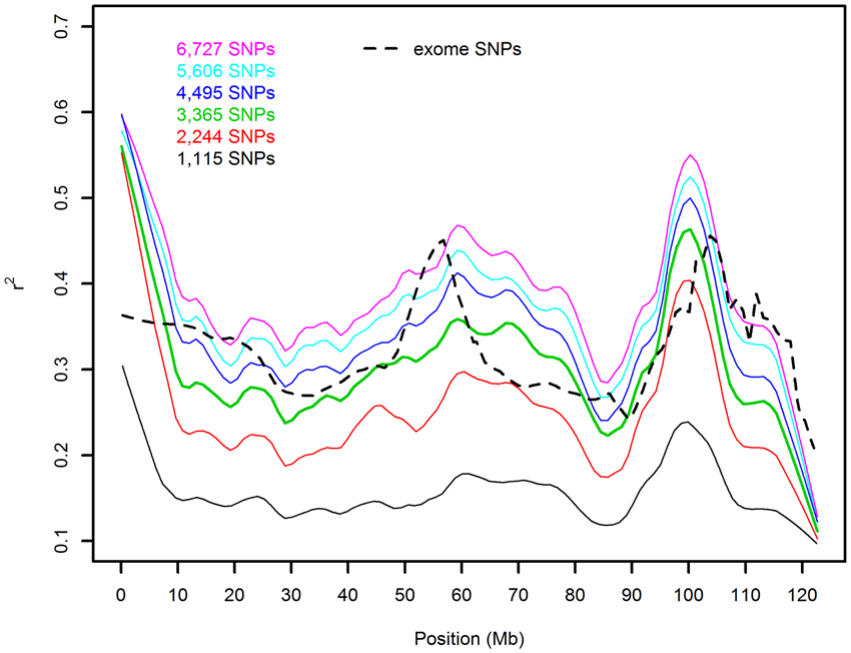
The effect of subsampling SNPs on r² relative to position. From the original 9541 SNPs on chromosome 1, random subsampling was performed, reducing the number of SNPs from 9000 to 1500 in 6 steps of 1500. In each step, 10 subsets were randomly sampled (without replacement). The number of SNPs that were polymorphic is depicted in the graph. At 4500 SNPs, WES and cHD had an equal number of informative tagSNPs (WES: 3310 SNPs, cHD: 3365 SNPs). Lines are obtained with LOWESS (locally weighed scatterplot smoothing).

A direct comparison of the amount of LD between subsequent SNPs (expressed as r²) for cHD and WES revealed that, for the majority of regions, the r² was higher for cHD (Fig. 2(c)), while the relation between distance between SNPs and r² was relatively similar and in agreement with expectations (Fig. 2(d))^17^. The former seemed to be largely related to the higher number of SNPs available for cHD because the median r² became identical when the number of SNPs was stepwise subsampled until they were similar for both techniques (Suppl. Table S2). In addition, a comparison of the r² over chromosome 1 demonstrated that, at that moment, WES had a higher r² on large parts of chromosome 1 (Fig. 3). These results clearly stress the effect of SNP quantity on r² ^18^.

Generally, within dog breeds, the r² tends to be very high for short distances, decreases quickly, but remains relatively high over a long distance^2^. For the Poodle, the r² was more than 0.6 at a distance of up to 5 kb, which is in the same range as the r² of 0.59, 0.62 and 0.52 at a distance of 0 to 10 kb that was found for the Golden retriever, Rottweiler and Newfoundland, respectively^19^. At a distance of 100 kb, the r² decreased quickly to values of around 0.2 while over a longer distance (e.g. for the Poodle on chromosome 1, r² of ≥0.1 at a distance of 700 kb), the LD remains above background (Fig. 2(d))^19^. In addition, even though for the majority of regions the number of variants for WES is lower than for cHD, the r² is not necessarily so (e.g. position 50 to 60 Mb on chromosome 1, Fig. 2(a,c)).

On a genome-wide basis, the r² was found to be both chromosome and breed-dependent (Suppl. Table S3, Suppl. Data S1) with the Poodles having the lowest LD (for n = 16: median WES = 0.17, median cHD = 0.23; for subsets of n = 6: median WES = 0.27, median cHD = 0.28), followed by the Labrador retriever (n = 6: median WES = 0.28, median cHD = 0.36) and the Golden retriever (n = 6: median WES = 0.36, median cHD = 0.40). For the Poodle, no comparison with other reports could be made while for the Labrador retriever and Golden retriever, results are in agreement with previous studies^2,6,7^.

### The relation between power and SNP position, SNP density and sample size

Power analyses, i.e. analyses that determine the probability to correctly reject the null hypothesis, were conducted to assess how power depends on the position of the causal SNP (within or outside target regions), the SNP density and sample size. Causal mutation signals were simulated according to a monogenic recessive phenotype (see methods). Based on an extensive preliminary analysis, detailed in Suppl. Data S2 and Suppl. Fig. S1, a window size of 10 SNPs on each side of the causal SNP, corresponding to a medial physical distance of ≈250 kb was used throughout these power analyses to detect the causal SNP based on the observation that close SNPs typically generate the significant association while distant SNPs increase the probability of spurious associations^20^. As the Poodle breed had the lowest r², we focused on this breed to assess the power for a within-breed association study. For all comparisons, results for simulations under H0 (see methods section) are provided in Suppl. Fig. S2.

**Figure 4:**
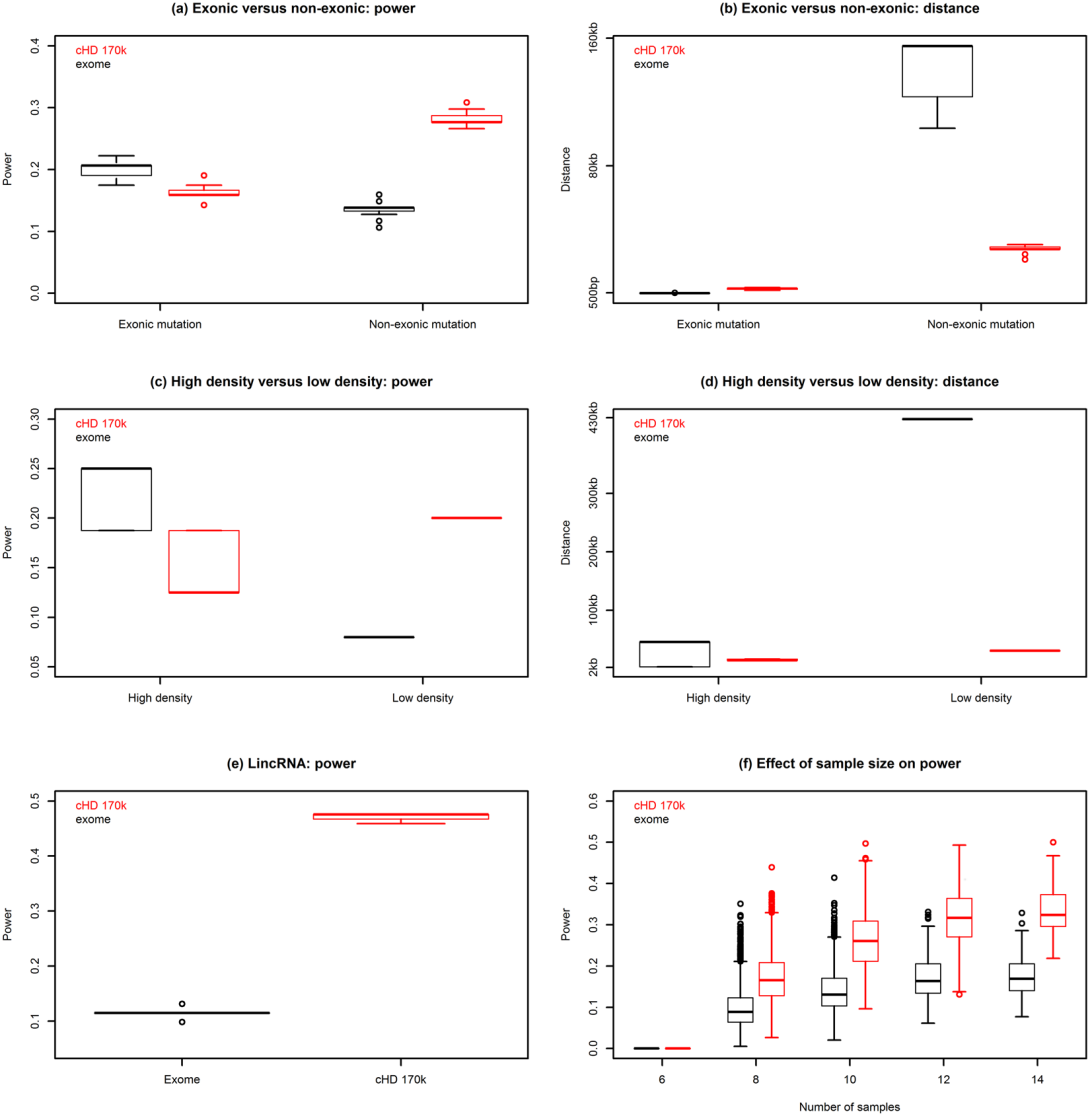
Power and distance between causal and tagSNPs for the exome-1.0 and canine high-density 170k array. (**a**) Boxplots showing the power to detect the association when a signal is located inside the target regions and outside the target regions. (**b**) Boxplots showing distance between the most significant SNP and the causal SNP when the signal is located inside or outside the target regions, respectively. (**c**,**d**) Boxplots showing power and distance to detect a non-exonic signal inside WES bins with a high informative SNP density (corresponding to the 85^th^ percentile or higher, threshold: ≥48 SNPs/Mb) and a low informative SNP density (corresponding to at most the 15^th^ percentile, threshold: ≤4 SNPs/Mb). (**e**) Boxplots showing power to detect a signal in long intergenic non-coding RNAs (lincRNAs). (**f**) Effect of sample size reduction on power to detect a monogenic recessive trait. Subsampling was performed stepwise, from 14 down to 6 samples and for each step, at least 20% of all possible permutations of samples were performed. The bottom and top of the boxplot represent the first (Q1) and third quartile (Q3), while the horizontal line in the boxplot represents the median. Whiskers represent 1.5 times the interquartile range (Q3-Q1).

A direct comparison for a sample size of n = 16, reveals that WES and cHD differ with respect to power to detect a signal inside and outside target regions (Fig. 4(a,b)). For a signal outside target regions, the median power for WES is 14%, while it is 28% for cHD and the median distance between the causal and the most significant SNP is 155 kb for the former and 28 kb for the latter. For an exonic signal, the median power was 21% for WES, while it was 16% for cHD. The median distance between the causal and most significant SNP was very short (217 bp) for WES, as expected, and 3 kb for cHD. For non-target regions, cHD has thus more power than WES (Fig. 4(a,b)) and the opposite is true inside target regions. For target regions, this power difference has to be seen in the light of the slight tendency for cHD towards an inflated type 1 error (Suppl. Fig. S2), indicating the true difference between WES and cHD might even be (slightly) bigger. While the inverse relation between power and distance can already be expected based on the LD-distance graph (Fig. 2(d)), this was also directly confirmed by evaluating the influence of increasing the distance between tagSNP and signal on power to detect this signal (Suppl. Fig. S3): when the distance increases, the power drops. Overall, the LD-distance and power-distance graphs (Fig. 2(d) and Suppl. Fig. S3) are remarkably similar and confirm that that high power goes hand in hand with a high r² ^21^.

Whereas the overall power results give a general indication, it is likely that power will also be relatively variable in different regions. To provide a more in-depth analysis, the power of WES to detect a signal in non-exonic regions was compared for regions with a high-density of SNPs (85^th^ percentile or higher i.e. ≥48 informative SNPs per 1 Mb bin) and a low-density region (15^th^ percentile or lower i.e. ≤4 informative SNPs per 1 Mb bin). In high-density regions, WES outperformed cHD (median power of 25% relative to 13%), while the opposite happened in low-density regions (Fig. 4(c,d)). As expected, the power to detect non-exonic signals is directly related with SNP density.

While the results indicated differences between EG-GWAS and GWAS with cHD, the possible effect of price differences and, as a consequence, differences in sample size has not yet been assessed. Pricing is however highly variable and depends on many parameters such as the design used (e.g. exome-1.0 or exome-plus), sequencing settings (read length, number of samples pooled for capture, sequencing depth aimed for), and even varies widely between countries and laboratories. In more detail, at our laboratory, GWAS prices range from €200 and €500 (depending on the choice of the array), while the price range for WES goes from a little under €500 up to €1000 per sample (depending on the WES design used). As such, the relative number of samples, expressed as GWAS:WES, that can be analyzed for the same price ranges from 1:1 to 5:1. To assess both the direct effect of sample size and indirect effect of price on power, the sample size was gradually lowered for each technique while simulating autosomal recessive non-exonic phenotypes. By comparing the power difference between 16 samples and lower sample sizes within each technique separately, the effect of sample size can be assessed. By comparing the power for two different sample sizes between the two techniques, corresponding to a GWAS:WES sample ratio of interest, the indirect effect of price on power can be estimated.

Firstly, when the effect of sample size is assessed for EG-GWAS, the median power remained relatively constant for 14 and 12 samples relative to the power with 16 samples (Fig. 4(f)). After that, it gradually decreased up until it became zero for 6 samples (3 cases vs 3 controls). The same result was obtained for GWAS using cHD data (Fig. 4(f)). The power varied more (i.e. increased distance between upper and lower whiskers of boxplots (Fig. 4(f)) when the sample size decreased from 14 to 12 samples, indicating that the power is influenced by which samples were retained. As expected, sample size can influence power: whereas small differences do not necessarily influence power a lot, large differences will for sure. When the price difference between the two techniques is considered, it is clear that in the 1:1 situation, the power difference is identical to the various situations detailed before (Fig. 4(a,c,e)). When the price difference increases gradually from 1:1 to 5:1, the power difference between GWAS and EG-GWAS will increase, as can be seen in Fig. 4(f). The maximum difference in sample size that could be evaluated in this case was a 2.7:1 ratio. As such, the difference in price should be considered when choosing for GWAS or EG-GWAS. At present, this means that even though sequencing prices have dropped rapidly, for a given budget, more samples can be analyzed on GWAS and the power will likely be higher, especially outside target regions.

### Proportion of the genome in high LD using EG-GWAS

We estimated the fraction of the genome that is in high LD with WES variants. Earlier analyses demonstrated that WES outperforms GWAS within targeted regions. For the exome-1.0, this means 53 Mb is highly accessible. As we determined that r² > 0.6 at a distance up to 5 kb, we hypothesized that within 5 kb of the target regions, the power likely remains high(er) for WES. By combining the target region size and adding 5 kb on both side of target regions with variants, we estimated that WES has a high LD for 12.6% of the genome which is a more than 6 times extension of the exome part (≈2%) directly targeted by WES. The portion under high LD varies from 6% to 22% per chromosome. As a consequence of the target definition, the regions that are likely under high LD contain all exons and their regulatory sequences including promoter regions of genes or *cis*-acting regulatory elements, which typically regulate gene transcription by functioning as binding sites for transcription factors.

### lincRNAs and EG-GWAS

To assess directly whether an EG-GWAS-based approach has power to detect signals in real regulatory regions that are off-target for the exome-1.0 design, a phenotype was simulated in regions corresponding to long intergenic non-coding RNAs (lincRNAs) as described in the recent updated dog annotation^22^. Phenotypes were simulated within lincRNAs on chromosome 1 that are by definition located outside the WES target regions. In WES, the median power was 11% with a median distance of 165 kb between the signal and the tagSNP. For cHD, the power was 48% with a median distance of only 23 kb (Fig. 4(e)). The difference of over 100 kb in distance accounts for the reduced power of WES to identify variants in these regions.

### Real phenotypes: the identification of genetic variants responsible for traits shared across breeds

Many dog breeds share mutations causing common traits and several phenotypes have been mapped by using an across breed mapping approach^8,16,23–25^. Here, we used the coat patterns within 3 breeds to compare EG-GWAS and cHD for their capability and precision to detect loci associated with hair length (long hair is fixed in Golden retriever and Poodles) and furnishings (fixed in the Poodle breed)^15,16^. Given the fixation status of these traits, these well-defined phenotypes are relevant to investigate the mapping precision of an EG-GWAS approach across breeds. Furnishings is a characteristic pattern of a moustache and eyebrows and is determined by the presence of a 167 bp insertion in the 3′UTR of the *RSPO2* gene, which is located at chromosome 13 and is inherited in an autosomal dominant manner^16^. Hair length is associated with an autosomal recessively inherited G > T variant in the *FGF5* gene^15^. For each phenotype, two analyses were performed. Firstly, using the entire dataset of 28 dogs, the p-value and the distance between the closest significant SNP were compared for EG-GWAS and cHD. Next, the sample size was stepwise reduced to find the minimal sample size necessary to identify a significant association.

A detailed overview of the results and Manhattan plots are provided in Table 1 and Suppl. Fig. S4, respectively Significant associations nearby and at the respective causal indel and variant were detected for both phenotypes. For hair length, EG-GWAS directly identifies the causative mutation (Bonferroni-adjusted p-value = 1.87e-06)^15^. The variant was also ranked number 1 in the list of most significant SNPs. The causative variant identified by EG-GWAS is within exon 1 of *FGF5*, in which a highly conserved Cys is changed to Phe (Cys95Phe)^15,16^. In comparison, for cHD-based GWAS, the best SNP is significantly associated (Bonferroni-adjusted p-value = 0.002) but ranked 32^th^ and is located 200 kb away from the causative variant in exon 1 of *FGF5*. For furnishing, EG-GWAS identifies associated variants (Bonferroni-adjusted p-value = 1.85e-05, ranked 2^nd^ and 3^rd^ in the list of most significant SNPs) close to the causative variant. The closest associated variant (ranked 3^rd^) is within the 3′UTR of *RSPO2* at 1.3 kb away from the causative indel^16^. For GWAS with cHD, the best significantly associated SNP (Bonferroni-adjusted p-value = 4.6e-07) is ranked first and resides 25 kb away from the causative indel. For EG-GWAS, 6 dogs per breeds (total sample size = 18) was the minimum to identify significant associations for both traits^2,8^. For cHD, the minimum total sample size was also found to equal 18 for furnishing, but for hair length, the sample size of 28 was necessary. Although these results are expected as both causal variants are located in exonic regions, they clearly illustrate the high power of EG-GWAS to discover associated genes and also evidence the practical usability of EG-GWAS for traits shared across breeds without having to predetermine linked loci first and, given the low sample size, at a reasonable cost.

**Table 1:** Characteristics of the closest significant SNP for each method and each phenotype. For each method, the SNP location, its p-value and rank are provided for the tagSNP that is closest to the causal mutation. The columns labelled “Genotype distribution of cases” and “Genotype distribution of controls” detail the number of times a specific genotype (AA, AB or BB for di-allelic markers with alleles A and B) occurred for cases and controls, respectively. Whereas EG-GWAS was each time closer to the causal mutation, its result was less significant for furnishing because that tagSNP was only called in 8 out of 16 cases. This higher variability in call rate was expected based on earlier reports^5,10^ (see methods). Nevertheless, for EG-GWAS, the genotypes of cases and controls are perfectly separated whereas for cHD, there is an overlap of 1 sample for furnishing and 5 samples for hair length. Manhattan plots are presented in Suppl. Fig. S4.

Phenotype	Technique	SNP	P-value	Genotype distribution of cases (AA/AB/BB)	Genotype distribution of controls (AA/AB/BB)	Distance	Rank
Hair length	EG-GWAS	chr32:4509367	1.87e-06	6/0/0	0/1/17	0	1
	cHD	chr32:4299533	0.002	4/2/0	0/3/19	200	32
Furnishing	EG-GWAS	chr13:8611728	1.858e-05	8/0/0	0/0/12	1	3
	cHD	chr13:8635445	4.601e-07	1/0/15	12/0/0	25	1

EG-GWAS = Exome-guided genome-wide association studies.

cHD = canine High-Density SNP array.

P-value = Bonferroni corrected p-value of closest significant SNP.

Distance = Distance between closest significant SNP and causal variant (in kb).

Rank = position of the SNP when all SNPs within a method are ranked from most significant to least significant.

## Discussion

WES allows a direct assessment of the protein-coding regions inside the genome^26^. In the dog, as in other species, exome sequencing has been very successful in the identification of disease causing mutations for a range of dominant and recessive Mendelian disorders^27–31^. While WES is an efficient method to analyze directly the nucleotides of a patient’s DNA to discover the genetic cause of Mendelian phenotypes, we investigated the possibility to extend the paradigm of WES in the dog by evaluating the potential of WES to detect LD-based associations.

Based on the definition of association studies, the two prerequisites to conduct efficient analysis are the presence of tagSNPs and LD between the tagSNP and the causal variant. Fundamentally, there is a clear difference between GWAS and WES with respect to these tagSNPs. Typically, in GWAS, a predefined number of more or less equally spaced, informative tagSNPs are interrogated^8^. For WES, the number of variants is unknown *a priori* and less limited because, theoretically, every targeted base pair can vary. Important is also that these variants are not selected to fulfill prespecified criteria, except for a bias towards the target regions. Based on this description, the more unequal distribution of SNPs and the observed association with gene density can be expected for WES. This also explains why from all SNPs detected, only a small fraction was in common (2.1% for EG-GWAS and 1.4% for cHD).

Although the design of the canine 170 K SNP array ensures the presence of equally spaced SNPs, it does not guarantee informativeness of all SNPs i.e. predefined SNPs may not be polymorphic (minor allele frequency (MAF) > 0.05) in the breed and/or population being investigated. In addition, rare SNPs (MAF < 0.01 or < 0.05) are sometimes removed as part of the quality control^32^. As an example, in this study, from the 170k SNPs, subsets of only 59%, 66% and 71% of SNPs were polymorphic (MAF > 0.05) for the population of Golden retrievers, Labrador retrievers and Poodles, respectively. For WES, genotypes can be determined at every position that is sequenced at a sufficient depth and with sufficient quality and that for each individual. This enables the use of all SNPs, even those that occur at low frequencies. Especially when rare variants for common disorders are investigated, these SNPs are highly informative^33^. In that aspect, it should also be kept in mind that the detection of rare variants responsible for common disorders is typically one of the weaknesses of GWAS, as rare variants are less likely to be well-associated with GWAS tagSNPs. This is a consequence of the direct relation between r² and allele frequency: by imposing MAF restrictions on the tagSNP, the allele frequency of the causal SNP also has to be within the same range to have a high r² ^34^. This problem increases even more when *de novo* variants are investigated as these will always remain undetected with GWAS^35^. So, while traditional GWAS generally will be less expensive, allowing more samples to be analyzed for the same cost, these limitations of traditional GWAS have to be taken into account.

Overall, we have demonstrated in this study that WES is partly capable of detecting associations with causal SNPs for monogenic traits outside target regions. The power to detect these associations depends on the combined effect of several factors. Whereas an increased SNP density generally results in both a higher power and a higher r², the power also depends on the region the causal SNP is located in. For some regions, a high r² can be attained, even with a limited number of SNPs, while for others, a relatively high density and/or a close distance between causal and tagSNPs does not necessarily result in the highest power. These unexplained differences between the different signal categories (exonic, non-exonic, lincRNA) might be (partially) related to underlying differences in gene count, GC content and several other sequence features that have been shown to be associated with r² ^36^.

This study is unique and highly informative by its design as experimental data from both WES and GWAS SNP chip genotyping was available for 28 dog samples from 3 different breeds. As a consequence, GWAS and WES could each time be directly compared. Our results revealed that generally, WES outperforms GWAS inside target regions, while the opposite happens for non-target regions for monogenic traits. As complex disease loci often fall outside these protein-coding regions^14,37^, cHD is then the method of choice for complex trait mapping. However, cancer-driving mutations in promoter regions, which regulate gene expression, are located nearby exons and might be identified through LD by EG-GWAS^38^. In addition, newer designs such as the exome-plus have left the path of a pure protein-coding focus, resulting in exome designs that combine protein-coding regions (including UTRs) and non-protein coding regions (lincRNAs, miRNAs, antisense transcripts)^5^. For example, 82% of the 295 lincRNAs tested in this study have an at least partial overlap with the target regions of the exome-plus, hence, the power is expected to be far higher in that case^5^.

Whereas we analyzed the power for monogenic traits in three different categories (exonic, non-exonic, lincRNA), it is difficult to predict in which category a genetic study will fall and thus what the exact power of a study will be because generally there is no prior knowledge on the causal variant^36^. Overall power estimates either way suggest the dog should generally be well-suited to detect associations^2^. While power calculations for a wide range of inheritance models are beyond the scope of this comparative paper, we do provide the necessary r² estimates in supplement (Suppl. Table S3) to make power calculations based on the relation between the non-centrality parameter λ and r² much more feasible^21^. While the used study design was optimal in terms of case-control balance and by assuming a monogenic, highly penetrant trait, additional choices might still improve the power to detect associations. Firstly, as the power depends strongly on the SNP density of a specific region, choosing novel and bigger WES designs that target a larger region of the genome will likely result in a higher power^5,10^. Whereas we estimated that EG-GWAS with the exome-1.0 outperformed GWAS with the classical cHD array for 6 up to 22% of the genome (depending upon chromosomes), it is clear that a similar analysis for the exome-plus (target size of 152 Mb) would result in far higher estimates. The same applies for novel SNP array designs with higher density that will be developed. However, increasing SNP density does not necessarily always result in an increased power: as demonstrated for several regions (e.g. chromosome 1: position 50–60 Mb), a similar or even higher r² can sometimes be reached with a smaller number of SNPs. Secondly, we deliberately focused in this study on the Poodle breed for the power analysis. This breed had the lowest r², indicating that the association between causal and tagSNPs will generally be lower compared to other canine breeds. For the majority of other breeds, the LD is more extensive (as shown for the Golden and Labrador retriever), indicating that the power will in general also be higher^2,6,7^. Additionally, computational methods like genotype imputation have recently been shown to be both feasible and accurate in the dog and will likely improve mapping efforts in the near future^39^. Genotype imputation is a method that requires a reference panel to predict additional genotypes, based on a far smaller number of actually genotyped variants^39^. It thus has the potential to tremendously increase the number of variants. It should however be stressed that the quality of the imputation depends strongly on the reference panel and that especially rare variants can be difficult to impute^39^. As such, imputation should always be performed taking these limitations into account.

Given the population history of the dog, a multi-breed approach has been shown to be highly amenable, if used properly^8,16,23–25^. The benefits of such an approach were also evidenced in this study. For two phenotypes, fixed within breeds, a mapping approach across breeds was adopted. Firstly, it allowed us to restate that this approach is highly powerful, as previously described^8^. Secondly, it also demonstrated practically what can be expected for an EG-GWAS or GWAS approach for an exonic causal variant: for both phenotypes investigated, EG-GWAS had a high mapping precision with significant SNPs corresponding directly to or very close to the causal variant and the minimum sample size might be slightly lower. It has to be emphasized nevertheless that this does not necessarily implies subsequent steps will never be necessary: the first time EG-GWAS was used, the correct locus was significantly associated, but downstream WGS was necessary to identify the causal inversion due to its non-exonic location^11^. As a note of warning, especially for across breed association studies, precautionary measures to avoid spurious associations due to population stratification have to be taken^40^.

Whereas the focus lies here on the dog, we hypothesize the use of EG-GWAS in other species as well because the prerequisites are fairly limited. As WES designs are increasingly available for a wide range of species, the main limitation is the amount of LD in the genome. For both the cow (*Bos taurus*) and the domestic pig (*Sus scrofa*) WES designs have been developed^41,42^. However, in both species, the LD does tend to be lower^43,44^. These results suggest that it is likely that EG-GWAS will still be possible, however, sample size estimates will probably have to be increased to get significant results. This does seem to stress again that the dog is rather unique and highly amenable for these approaches.

To conclude, we show that WES SNPs discovered during sequencing can be used as tagSNPs to detect LD-based associations in a significant part of the canine genome that is not solely restricted to exons. As such, while WES is often utilized to detect causal SNPs directly, we conclude that it can also be used to detect causal SNPs indirectly, even when these causal SNPs reside outside target regions (promoters, transcription factor binding sites, enhancers, non-coding genes). The power to detect associations varies with SNP density and the region the causal SNP is located in, as demonstrated for autosomal recessive phenotypes. In general, WES outperforms GWAS inside target regions, while the opposite happens for non-target regions. Specifically for the exome-1.0, given its focus on protein-coding regions, this implies that cHD is the method of choice for complex disorders. Overall, we introduce here the concept of EG-GWAS based on real datasets, and provide hands on instructions and examples on how to carry out EG-GWAS-based genotype to phenotype analyses.

## Methods

### Samples

Blood and tissue biopsy samples from dogs were collected by a network of veterinarians through the French Cani-DNA biobank (http://dog-genetics.genouest.org), which is part of the CRB-Anim infrastructure [ANR-11-INBS-0003]. The work with dog samples was approved by the CNRS ethical board, France (35-238-13) for UMR6290 and dog owners consented to the use of data for research purposes anonymously. All methods were carried out in accordance with relevant guidelines and regulations. A paired study design was adopted in which the same 28 samples (from three breeds of which 16 were Poodles, 6 Labrador and 6 Golden retrievers, respectively) underwent both WES and GWAS genotyping.

### Genotyping

Genotyping was performed with the cHD 170k whole-genome genotyping BeadChip at the Centre National de Génotypage (CNG; Evry, France)^12^.

### Exome sequencing

#### Library construction

Dog exome libraries were generated from standard indexed Illumina libraries using a custom Roche/Nimblegen solution-based capture library (120705_CF3_Uppsala_Broad_EZ_HX1) following the protocol provided. Index-specific hybridization enhancing oligos were used.

#### Sequencing, Alignment and filtering

After capture, indexed libraries were pooled separately into groups of eight. Pools were run on an Illumina HiSeq2000 platform, with a target coverage of 60× depth to ensure maximal coverage of the 53 Mb targeted by the exome-1.0 WES design and high quality variant discovery. The reads were aligned to the CanFam3.1 reference genome^45^ using BWA 0.5.9. Duplicate reads were marked with Picard (http://broadinstitute.github.io/picard/), and the data were sorted. Using the GATK 3.5-0, local realignments were generated and base quality scores were recalibrated per GATK Best Practices^46^.

#### Variant calling

GATK 3.5-0 was used to realign the reads around indels and HaplotypeCaller was used to call SNPs on the resulting BAM files. The resulting VCF files were converted to PLINK (ped and map) format file using vcftools_0.1.13 and bi-allelic SNPs were considered (indels were not retained). In total, 83,869 unique SNPs were further used for Poodles, 66,809 for Labrador retrievers and 66,000 for Golden retrievers.

### LD analysis

Both the LD and power analyses were run from within R (version 3.3.1), using R-studio (version 0.99.902). The LD analysis was performed with Haploview 4.2^47^. The following filters were used: MAF of 0.05, Hardy-weinberg p = 0, exclude markers with >0.5 missing genotypes and less than 0.5 nonzero genotypes. The missing genotypes cut-off reflects the 5^th^ percentile of the % genotyped distribution of the 16 Poodles to account for the higher performance variability associated with sequencing relative to GWAS assays. This cut-off accurately removed outliers as all other tagSNPs had a success rate of >90%. An in-depth discussion on the rationale of the setting applied for the Hardy-Weinberg equilibrium test is provided in Suppl. Data S3^48–52^. The r² values between subsequent markers were retained and used in the downstream analysis to investigate the potential association with distance and chromosomal position. Minimum, Q1, mean, median, Q3 and maximum values were calculated for each chromosome per breed for cHD and WES separately. To allow an unbiased comparison of the r² between the three breeds, both raw r² values and r² values based on random subsampling (100 random combinations of each time 6 samples) were calculated for the Poodle. To evaluate the effect of the number of SNPs, random subsampling of the original 9541 cHD SNPs (on chromosome 1) was performed, reducing the number of SNPs from 9000 to 1500 in 6 steps of 1500. For each number of SNPs, the random subsampling and subsequent LD analysis was performed ten times. To determine the background LD, one SNP was randomly sampled from each chromosome, followed by calculating the r² between all pairwise combinations of these 38 unlinked SNPs. This random sampling was repeated 100 times and the median value of the obtained distribution was considered to represent the background r².

### Power analysis

H1: simulation of a signal, followed by simulation under H0. To assess the power, two approaches are generally used: starting from phenotypes, genotypes can be simulated, followed by association analyses. This is however computationally demanding and requires a correct creation of the LD structure of the genome. A second option that starts from the genotype to simulate phenotypes has the advantage that it immediately can be used from existing (GWAS or WES) genotype data and that it uses as such the real LD pattern of the dog breed^2,53^.

The second procedure was used: for each SNP, the genotype distribution was determined. Under the assumption of a fully penetrant autosomal recessive disease, one homozygous genotype is considered “affected”, while the two remaining ones are considered “healthy”. To mimic a balanced case-control design of 8 cases and 8 controls, only those SNPs were selected that followed this distribution (n = 91). At that moment, a perfect association between the genotypes for that specific SNP and the phenotype has been created. This causal SNP is next omitted from the association analysis itself. The reason is that a tagSNP is typically considered not to be the causal SNP itself: leaving it in would make it a direct method instead of an indirect method that has to rely on LD to detect the association. This would bias the power comparison as one method would have the causal SNP directly interrogated, while the other method would have to rely on LD to detect the association. This is repeated for all SNPs by walking over the chromosome and results in various but at the same time balanced combinations of the original samples. For each SNP that fulfilled the balanced criterion, the association analysis was conducted with PLINK (version 1.90b.45) by applying the Cochrane Armitage trend test to detect the association between phenotype and tagSNPs^54^. After each association test, the case-control labels were randomly permutated and the association test was performed again to evaluate the number of significant results under H0. To control the family-wise error rate (FWER), p-values were calculated with permutation (mperm procedure, 500 permutations based on the observation that adaptive permutation stopped often at lower values and the narrow dispersion of power values). P-values ≤ 0.05 were considered significant. This principle is used in all power analyses with slight adaptations.

### High density - Low density comparison

To enable an assessment of the effect of SNP density on power, regions with a high density of informative SNPs (corresponding to the 85^th^ percentile or higher, threshold: ≥48 SNPs/Mb) were compared to regions with a low density of informative SNPs (corresponding to at most the 15^th^ percentile, threshold: ≤4 SNPs/Mb). The signal was each time simulated by cHD SNPs outside the regions targeted by WES, but inside the high density/low density regions, respectively.

### Sample size

To assess the effect of sample size, 8 different sample sizes were evaluated by subsampling from the original 16 Poodle samples. The sample sizes tested were 14, 12, 10, 8 and 6 samples. For each step, all possible combinations of samples were calculated *a priori* and at least 20% of all possible combinations were randomly selected, the association tests were performed and the power was calculated as described before. In each step, a balanced design was mimicked, i.e. 7, 6, 5, 4 and 3 cases and controls, respectively.

### Distance effect

To evaluate directly the relation between power and distance between the causal SNP and the tagSNP, a subset of exome and cHD SNPs on chromosome 1 were selected to be used as tagSNPs based on the following filtering criteria: each tagSNP had to have a causal SNP (always a cHD SNP) at a physical distance between 1 bp and 20 kb, 50 and 300 kb, 500 and 800 kb and 1 to 2 Mb. Each time, the phenotype was simulated by the causal SNP and the association was tested using the tagSNP.

### LincRNA

To determine the power to detect signals in non-protein coding regulatory regions, cHD SNPs were identified that resided inside lincRNAs that did not overlap with any of the target regions on CFA1^22^. To retain a sufficient number of SNPs, a wider range of cases was allowed (i.e 4 to 12 cases), which resulted in a total of 61 unique SNPs that were used for the simulation of phenotypes.

### Identification of genetic variants responsible for traits shared among dog breeds

Two phenotypes were mapped as a proof of concept: furnishing and hair length^16^. The chi-squared allelic association analysis was performed with PLINK (version 1.90b.45) and Bonferroni corrected p-values were used^54^. For the first analysis, the entire dataset (n = 28) was used. As prior knowledge exists on the location of the causal variant, the closest significant SNP was selected and both the p-value and the distance to the causal variant were retained. The aim of the second analysis was to discover the minimum sample size. Stepwise subsampling was performed. For each sample size, 10 random combinations were selected and the corresponding GWAS and EG-GWAS datasets were used in an association study. The last sample size with at least 1 significant result was considered the minimum sample size. Across breed power studies were not possible due to the low number of SNPs that followed the expected fixed trait distribution.

### Data availability

The datasets analyzed during the current study are available in the Sequence Read Archive repository, under accession number SRP106684.

## Electronic supplementary material

Supplementary Information
