# Supplementary material for: An exome sequencing based approach for genome-wide association studies in the dog
Source: Sci Rep. 2017 Nov 15;7:15680. doi: 10.1038/s41598-017-15947-9 (PMC5688105; doi:10.1038/s41598-017-15947-9)
Supplement: Supplementary file 1 — Supplementary Information [file 41598_2017_15947_MOESM1_ESM.pdf]

An exome sequencing based approach for genome-wide association studies in the dog

Bart J.G. Broeckx[1*], Thomas Derrien[2], Stéphanie Mottier[2], Valentin Wucher[2], Edouard Cadieu[2], Benoît Hédan[2], Céline Le Béguec[2], Nadine Botherel[2], Kerstin Lindblad-Toh[3,4], Jimmy H. Saunders[5], Dieter Deforce[6], Catherine André[2], Luc Peelman[1‡], Christophe Hitte[2‡*]

[1] Laboratory of Animal Genetics, Faculty of Veterinary Medicine, Ghent University, Merelbeke, Belgium.

[2] Institut de Génétique et Développement de Rennes, CNRS-URM6290, Université Rennes1, Rennes, France.

[3] Broad Institute of MIT and Harvard, Cambridge, Massachusetts, USA.

[4] Science for Life Laboratory, Department of Medical Biochemistry and Microbiology, Uppsala University, Uppsala, Sweden.

[5] Department of Medical Imaging and Orthopedics, Faculty of Veterinary Medicine, Ghent University, Merelbeke, Belgium.

[6] Laboratory of Pharmaceutical Biotechnology, Faculty of Pharmaceutical Sciences, Ghent University, Ghent, Belgium.

[‡] Both authors contributed equally

Corresponding authors: Bart Broeckx, Bart.Broeckx@ugent.be and Christophe Hitte, Christophe.Hitte@univ-rennes1.fr

This file contains 4 supplementary figures, 3 supplementary tables and 3 supplementary data files. Captions and legends are also included.

Supplementary Figures

Supplementary Fig. S1. Effect of number of SNPs on power and distance for the exome-1.0 and cHD array. (a)-(b) Boxplots showing the power to detect the association and distance between the causal SNP and most significant SNP when a signal is located outside the target regions. (c)-(d) Identical, but for a signal located inside the target regions.

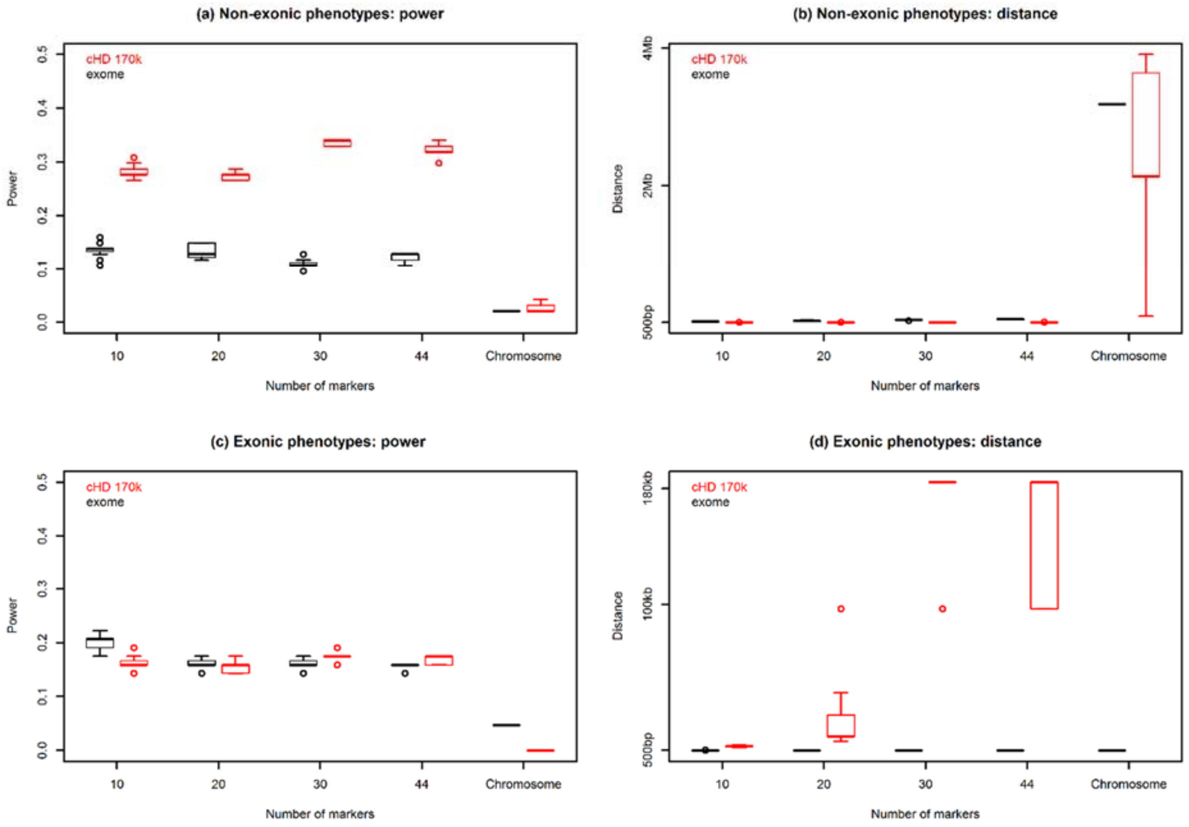

Supplementary Fig. S2. Type 1 error for the various power comparisons. From left to right, the median type 1 error was 0.03, 0.06, 0.05, 0.04, 0, 0, 0.04, 0.04, 0.03, 0.03. The type 1 error was similar for all comparisons, except for the exonic phenotypes where the type 1 error rate of cHD was twice the type 1 error rate of the exome.

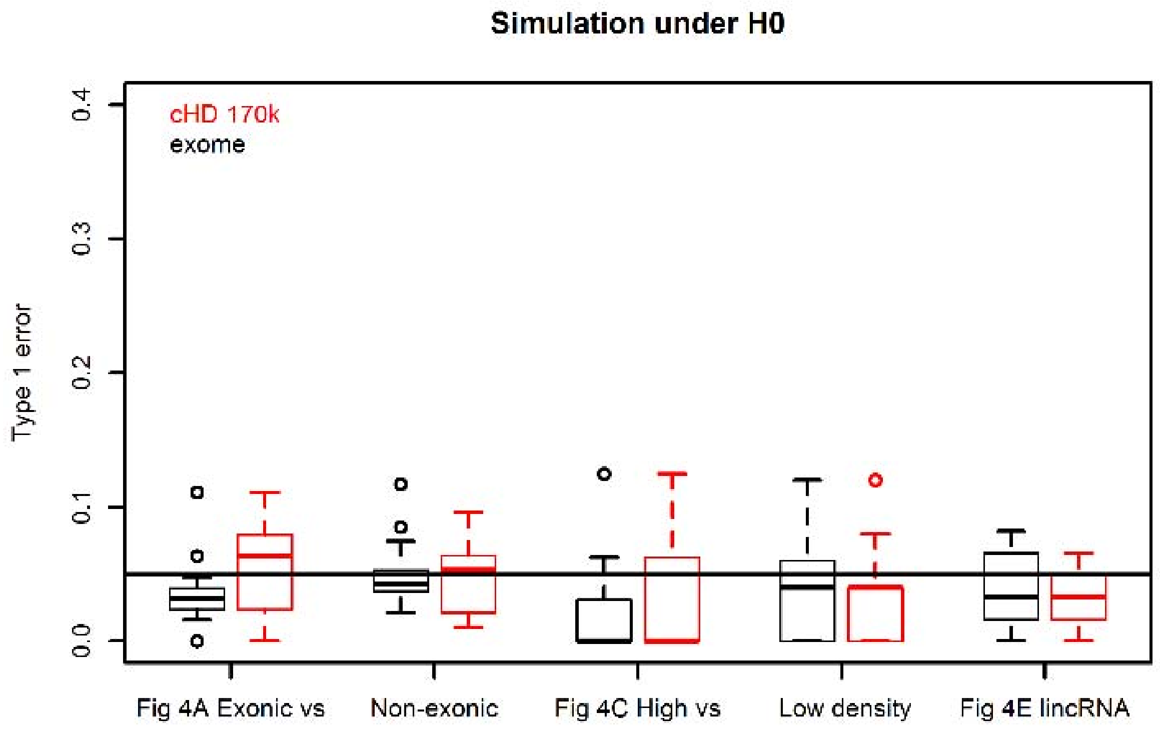

Supplementary Fig. S3. Effect of distance between causal SNP and tagSNP on power to detect the signal.

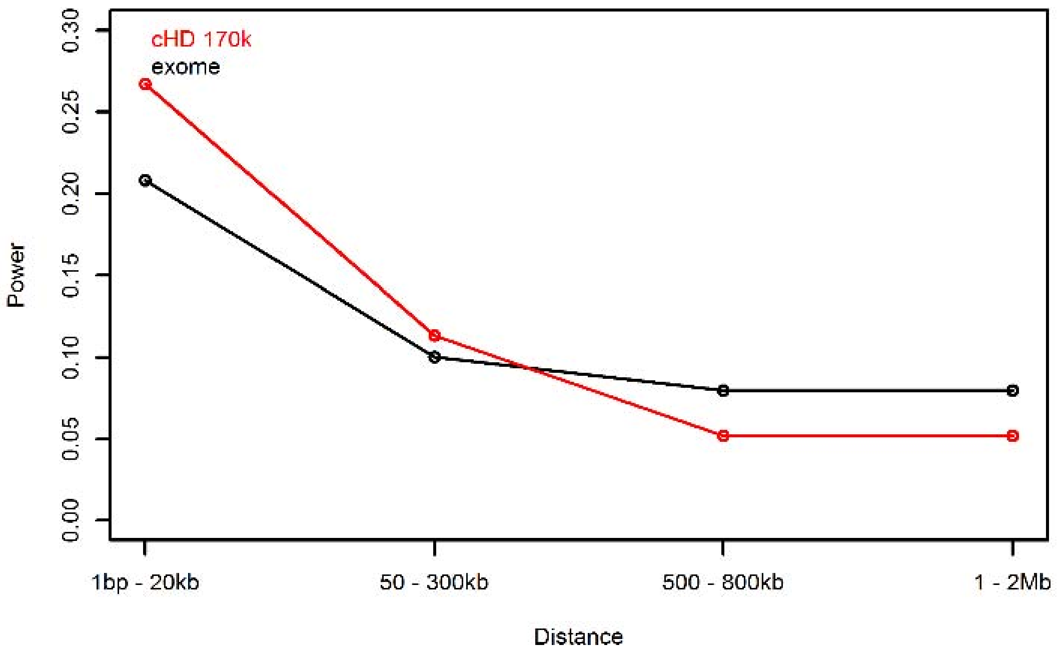

Supplementary Fig. S4. Manhattan plots for hair length and furnishing for EG-GWAS and cHD. A-B: Hair length, y = -log(10) p-values; C-D: Furnishing, y = -log(10) p-values; E-F: Hair length, y = Bonferroni-corrected p-value; G-H: Furnishing, y = Bonferroni-corrected p-value. I-L: reduced sample size, hair length, y = -log(10) p-values; M- P: reduced sample size, furnishing, y = -log(10) p-values. Left panel: EG-GWAS, right panel: cHD.

A.

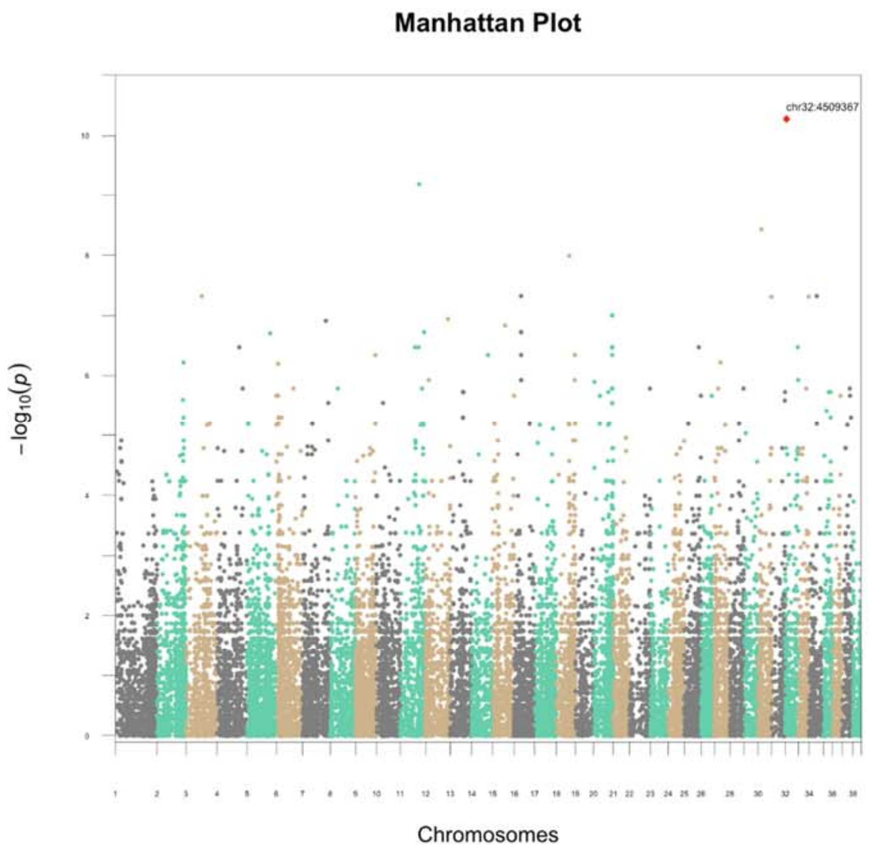

B.

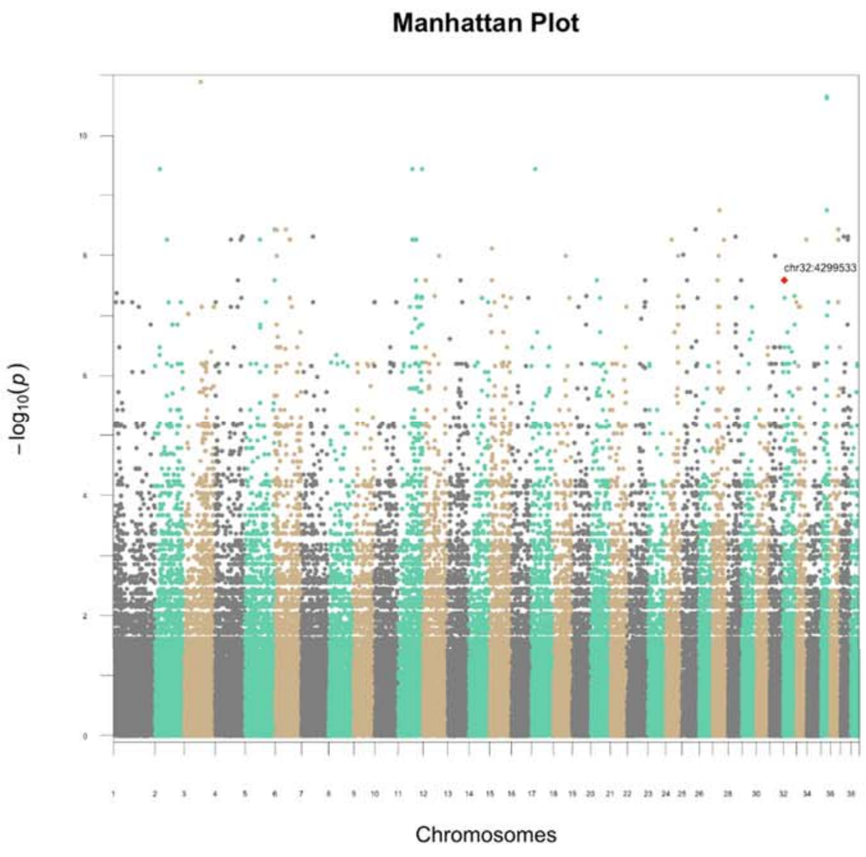

C.

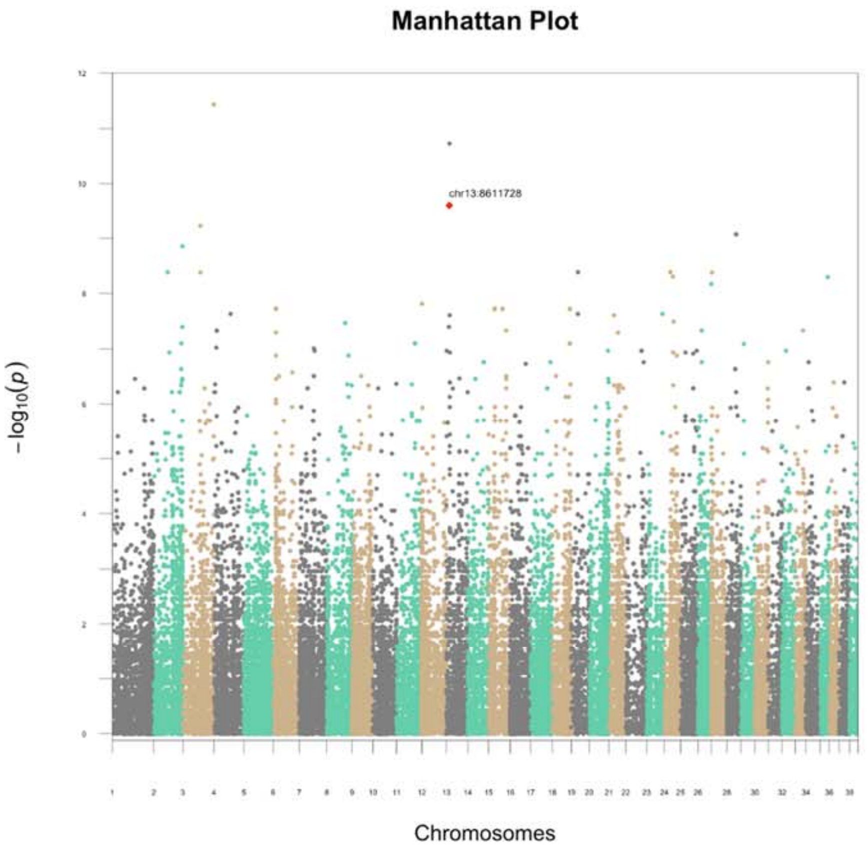
Manhattan Plot
chr13:8611728
$-\log_{10}(p)$
Chromosomes

D.

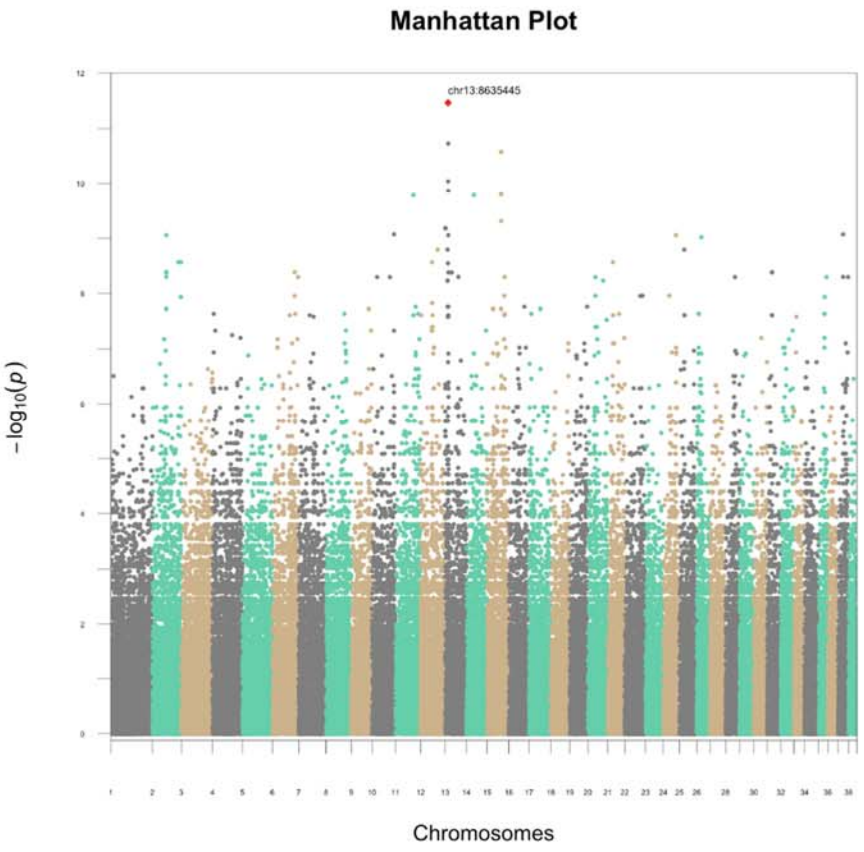
Manhattan Plot
chr13:8635445
$-\log_{10}(p)$
Chromosomes

E.

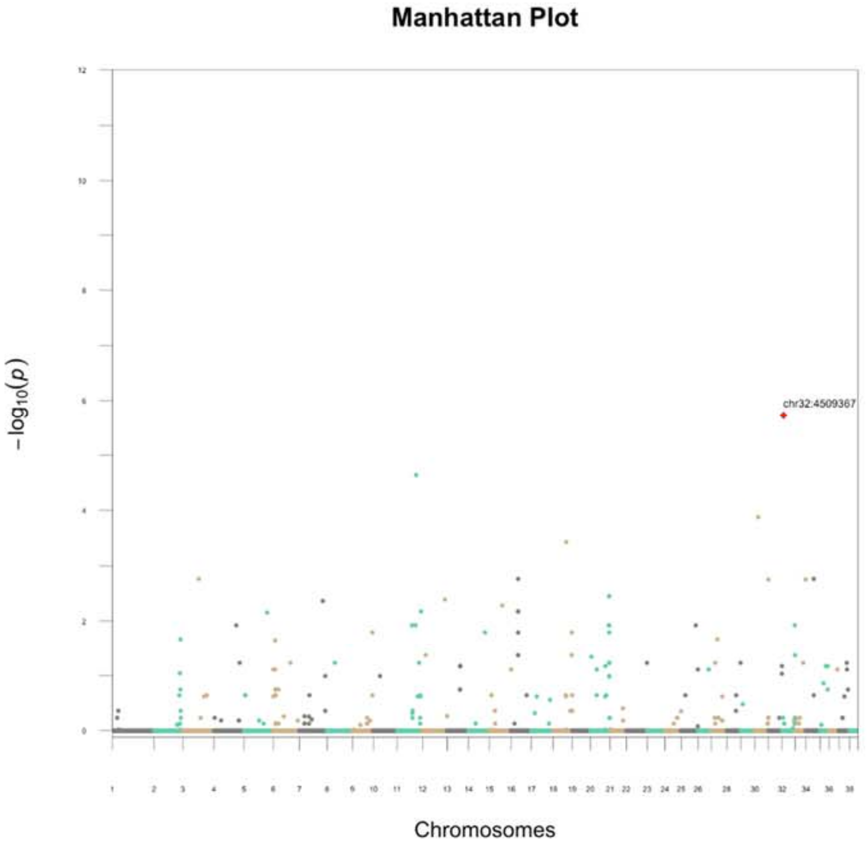

Manhattan Plot
$-\log_{10}(p)$
chr32:4509367
Chromosomes

F.

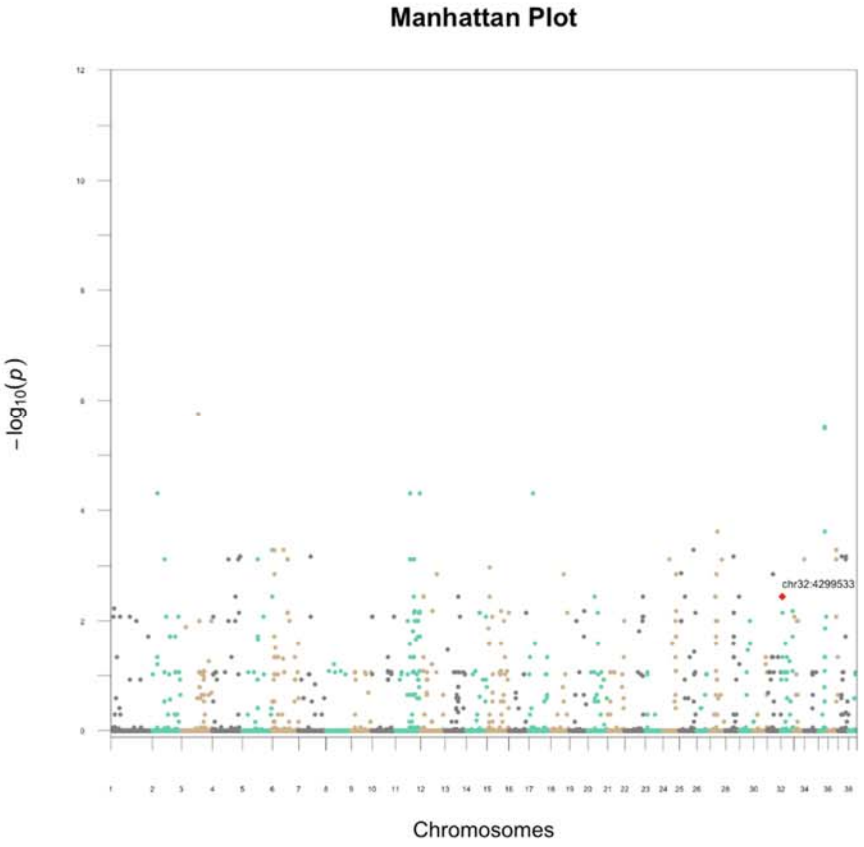

Manhattan Plot
$-\log_{10}(p)$
chr32:4299533
Chromosomes

G.

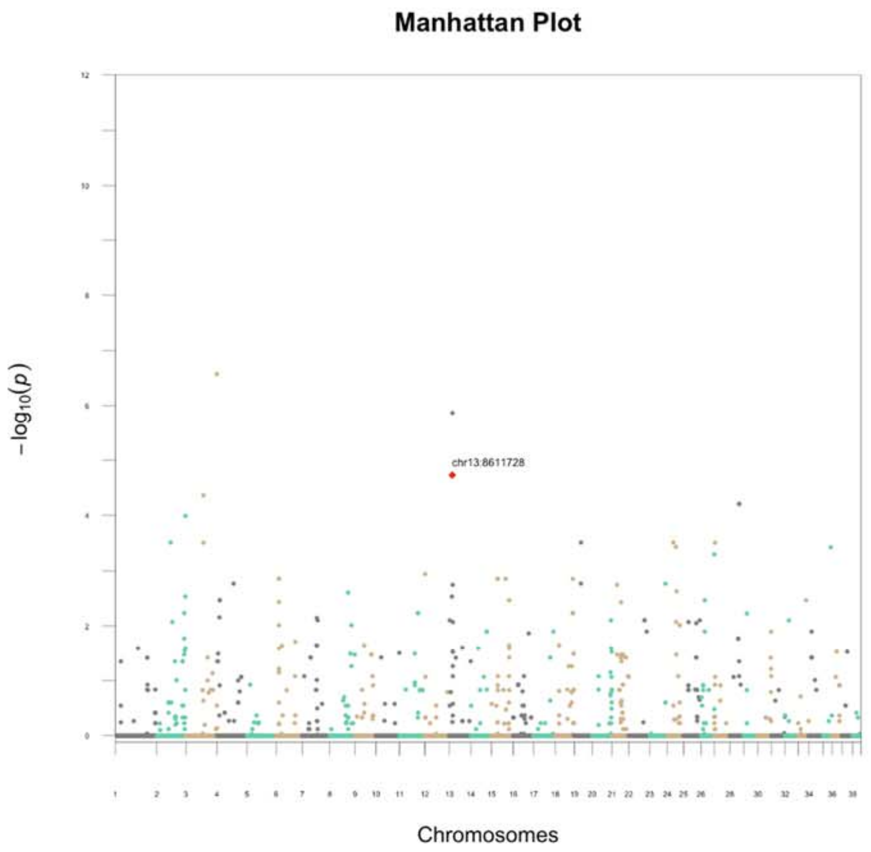
Manhattan Plot
−log10(p)
chr13:8611728
Chromosomes

H.

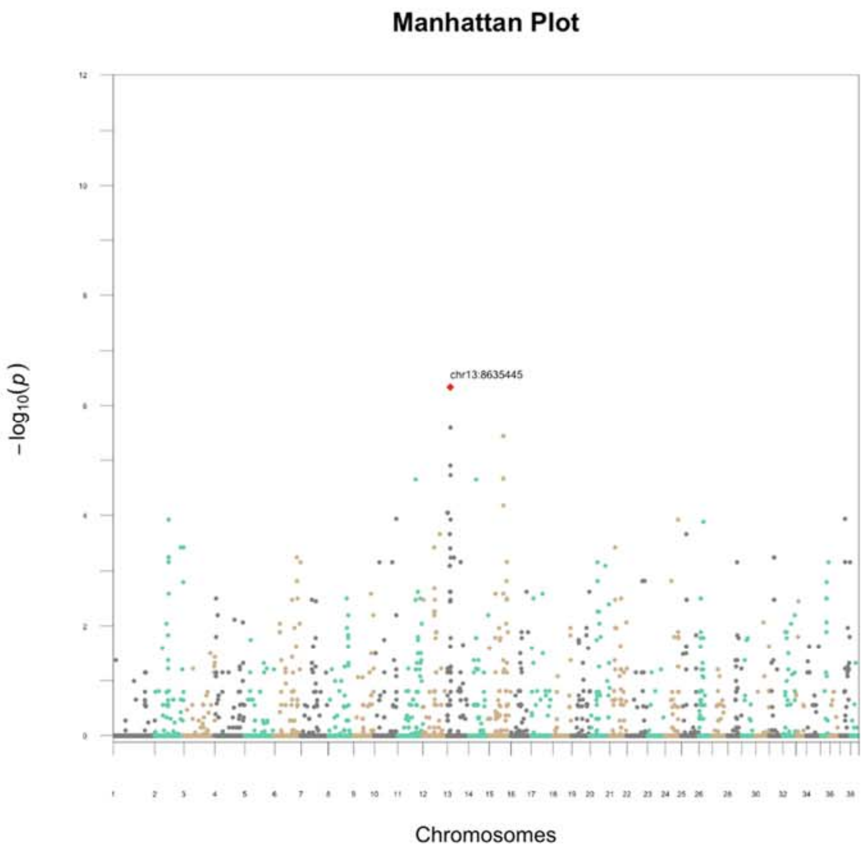
Manhattan Plot
−log10(p)
chr13:8635445
Chromosomes

I. sample size = 10 – 6 – 6

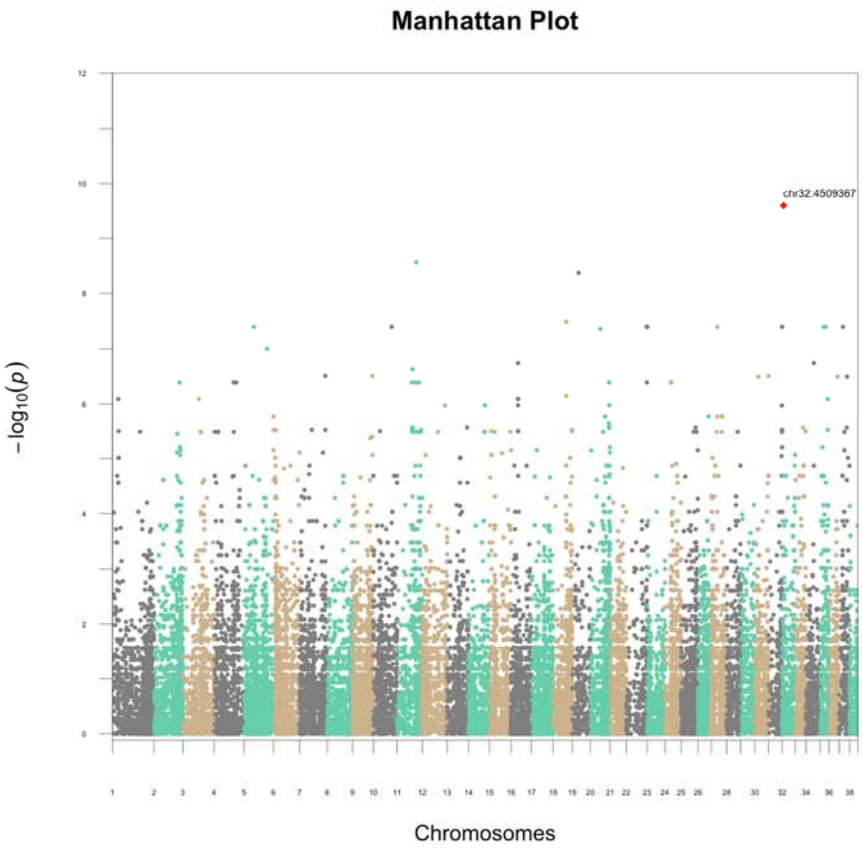

J. sample size = 10 – 6 – 6

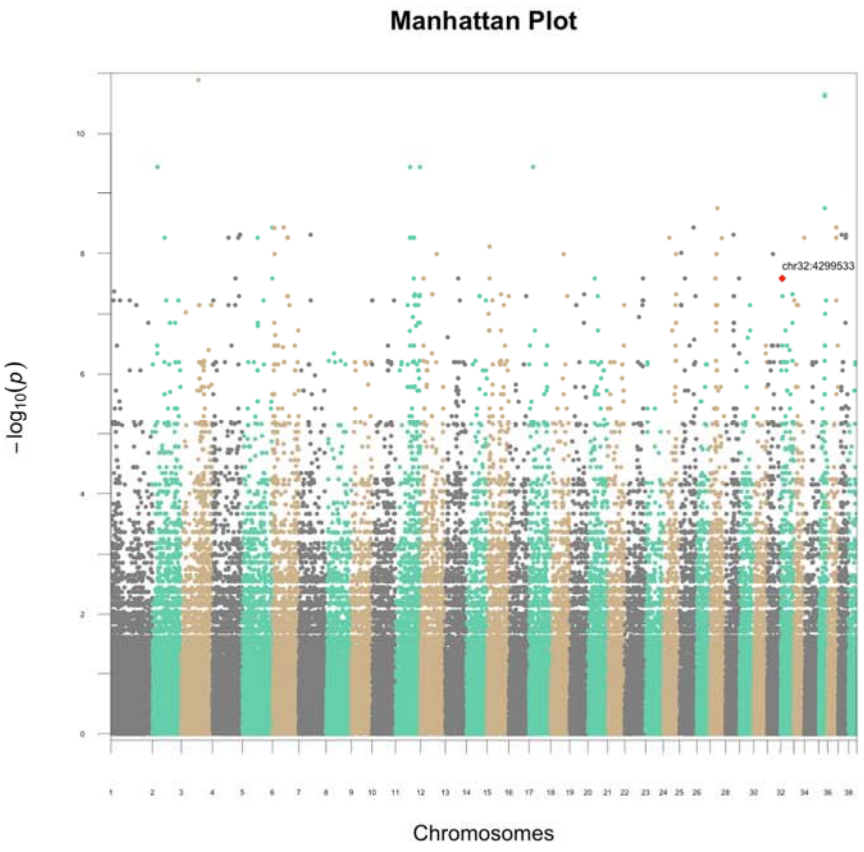

K. sample size = 6 – 6 – 6

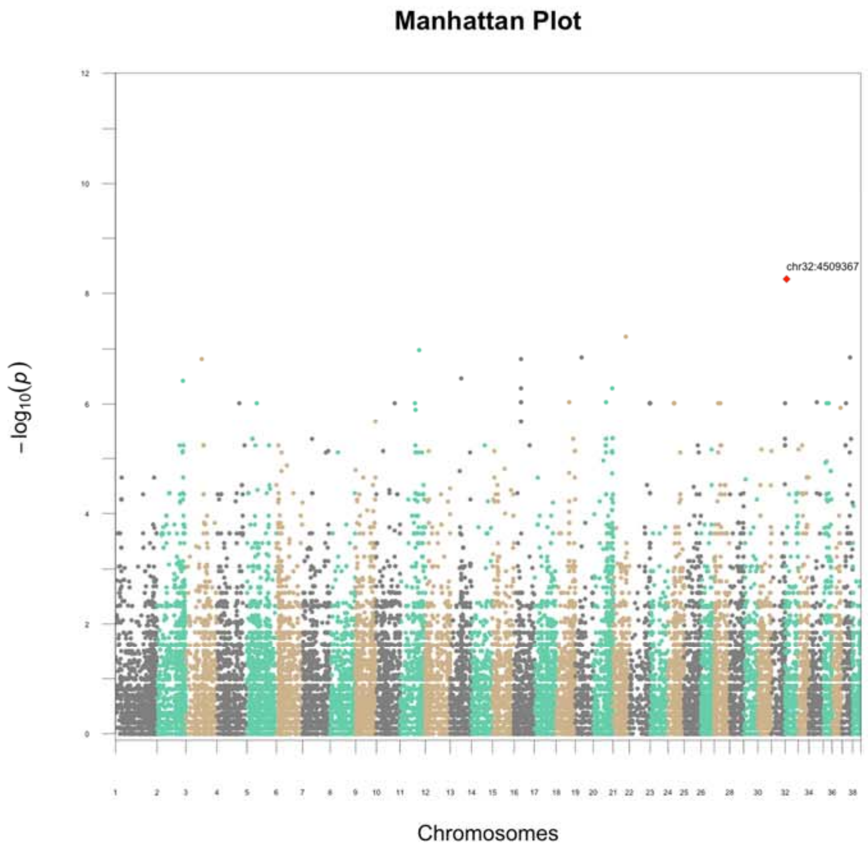

L. sample size = 6 – 6 – 6

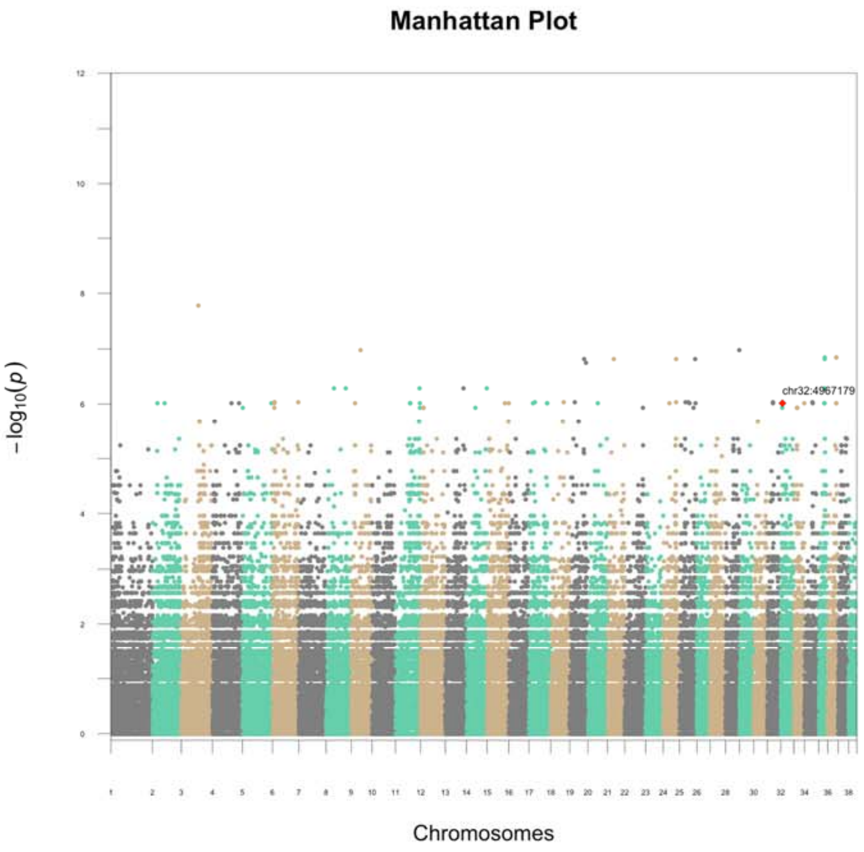

M. sample size = 10 – 6 – 6

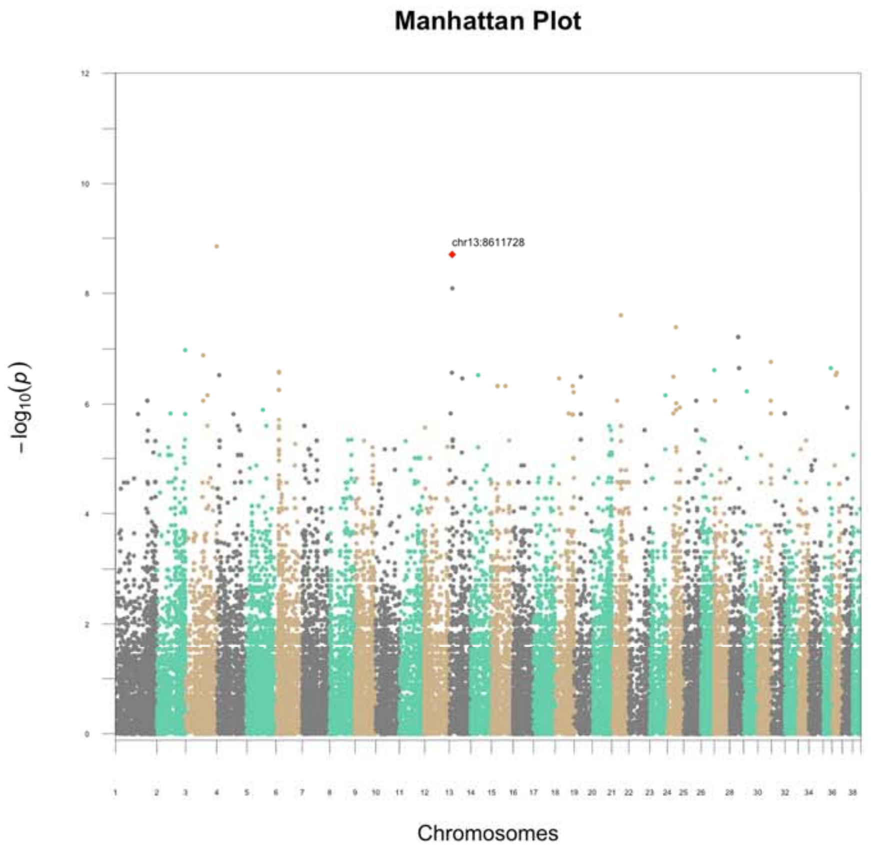

N. sample size = 10 – 6 – 6

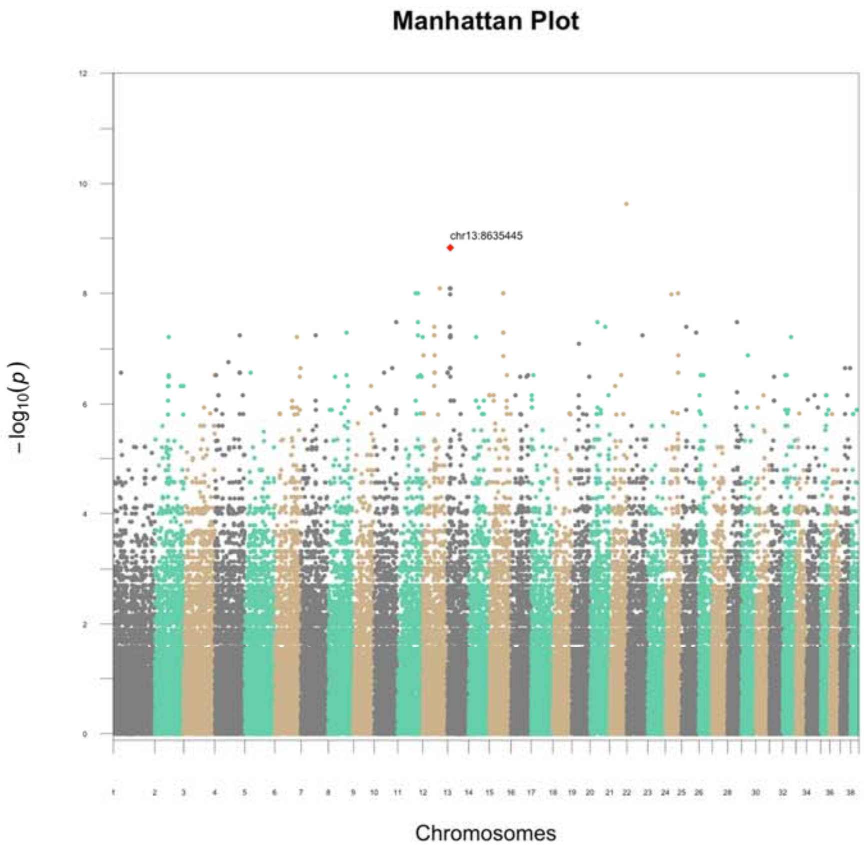

O. sample size = 6 – 6 – 6

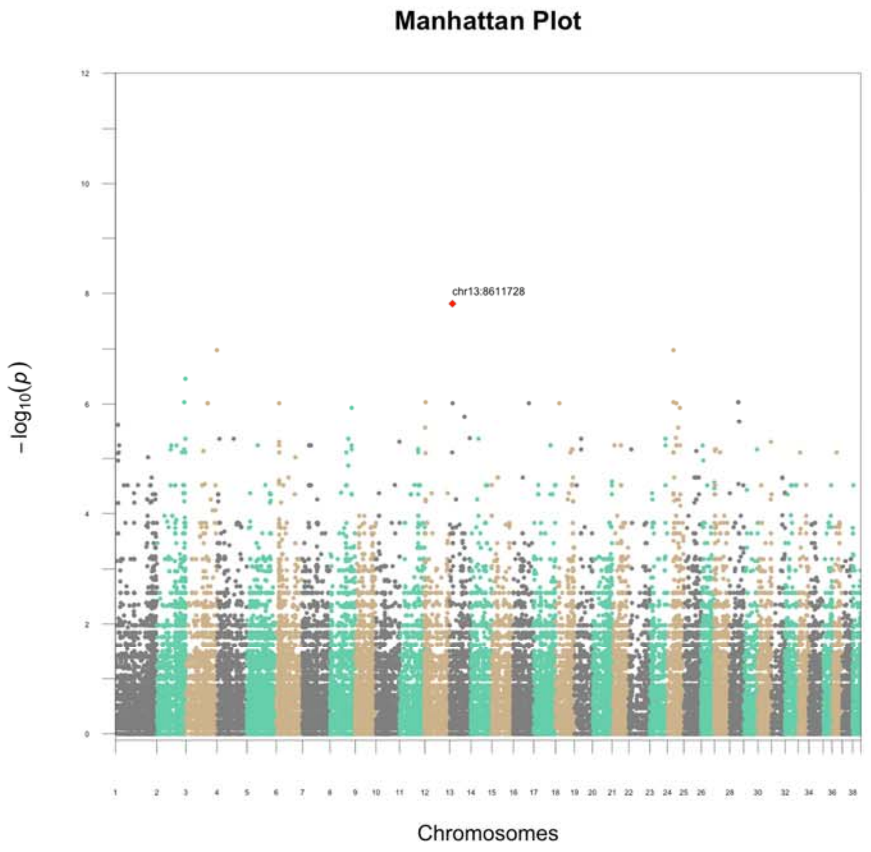

P. sample size = 6 – 6 – 6

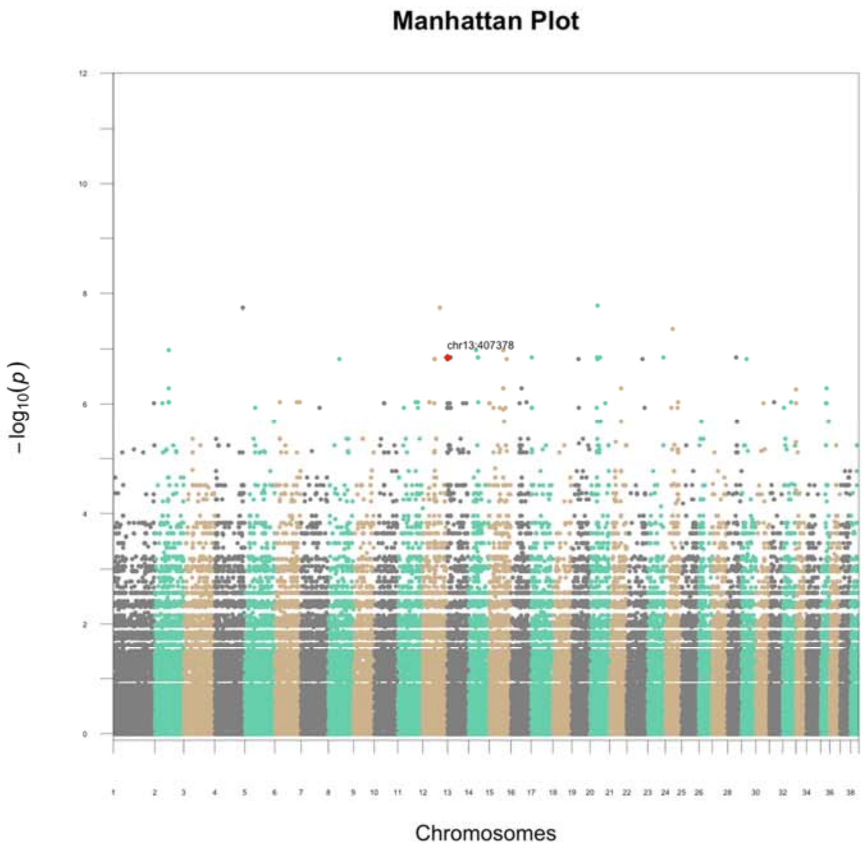

Supplementary Tables

Supplementary Table S1. Number of SNPs that passed the Haploview filters and distance distribution for the three breeds for exome and cHD

**Poodle**			cHD							Exome				
Chr	min	Q1	median	mean	Q3	max	min	Q1	median	mean	Q3	max	# SNPs exome	# SNPs cHD
1	17	8698	13400	18220	20850	487900	1	95	1446	36800	18330	1477000	3310	6732
10	18	8749	13530	18950	21150	405200	1	89	2190	41190	23430	2792000	1682	3653
11	17	8971	13940	20560	23100	901700	1	79	1839	51530	21340	2134000	1441	3614
12	20	8768	13470	17010	19980	215800	1	57	722.5	34660	11210	3030000	2088	4259
13	17	8483	13120	17090	19930	400300	1	55	1087	49720	17390	2268000	1271	3686
14	18	8892	13780	18300	21320	407400	1	104.5	2207	61540	33710	2215000	990	3329
15	17	9085	13830	18020	20990	208300	1	122.8	3062	55120	29530	1675000	1156	3556
16	17	8384	13110	17770	20190	596900	1	19	90	26460	3941	3412000	2245	3348
17	17	8193	12820	16650	19900	271300	1	79	1132	32280	15500	2163000	1980	3858
18	17	8578	13020	17610	19960	311000	1	46	287	19040	5847	2513000	2925	3163
19	22	8807	13130	16620	19470	256800	1	57	546.5	73200	26010	3294000	722	3231
2	17	8820	13440	19020	21090	1765000	1	81	1394	35010	16010	3484000	2428	4477
20	17	8601	13600	18450	21650	251500	1	127	1532	21610	9386	1653000	2682	3148
21	18	8505	13420	17130	20140	297200	1	50	263	25820	8939	2112000	1942	2963
22	17	8672	13020	16750	19730	383000	1	63	1034	85500	30180	3,00E+06	657	3658
23	17	8486	12950	16150	19220	188100	1	91.5	2708	54300	33980	2874000	959	3234
24	35	8253	12900	15840	19420	184500	1	65	1264	29820	13380	1614000	1595	3005
25	18	8185	12720	16250	18840	314700	1	45	408	30430	10420	1781000	1692	3173
26	17	7997	12890	17330	19430	724000	1	36	441	20890	9855	1664000	1862	2246
27	18	8658	13180	16500	19810	237600	1	34	309	22130	9394	1828000	2052	2767
28	18	8079	12620	16290	19230	245700	1	72	1841	32750	15030	2538000	1254	2525
29	27	8817	13020	16500	19820	195100	1	49.25	445	59470	33290	1610000	702	2529
3	17	8644	12980	16950	20010	371000	1	90	1869	55270	20170	3678000	1657	5420
30	17	8320	13100	16580	20280	298100	1	129	1886	29650	17910	1307000	1354	2424
31	24	8988	13160	17670	20420	1807000	1	61	830	42680	9923	2475000	933	2251

32	53	8904	13480	16960	20650	151900	1	65.5	623	45580	21090	2336000	847	2283
33	18	8684	13110	16310	19990	142000	1	32	250	30300	11810	2507000	1034	1923
34	19	8505	12630	15130	18510	272300	1	118.8	2338	56290	22400	2021000	744	2780
35	17	8001	12360	15130	18720	396200	1	44	250	31440	8418	1562000	837	1748
36	19	8682	12850	16280	19000	193200	1	141	749	40010	11660	1309000	750	1891
37	34	8539	13080	17390	19830	389200	1	122.5	1752	43160	16660	2684000	712	1769
38	17	8578	13040	15950	19220	429700	1	48	342	22660	5319	2121000	1052	1497
4	18	8634	13340	17240	20330	238800	1	109.2	2147	54920	22170	3138000	1606	5119
5	17	8548	13360	17480	20920	223100	1	115	1605	28480	13990	2328000	3017	5080
6	18	8481	13240	17480	20430	322800	1	63	987.5	27610	11140	2846000	2774	4418
7	18	8584	13160	17200	19940	452400	1	124	2716	42600	24210	4290000	1900	4702
8	17	9152	14350	18870	22390	355500	1	21	202	34330	10640	2552000	2163	3917
9	39	8756	13680	18700	22000	471200	1	131	1624	20310	11540	1059000	3000	3263

**Golden Retriever**			cHD							Exome				
Chr	min	Q1	median	mean	Q3	max	min	Q1	median	mean	Q3	max	# SNPs exome	# SNPs cHD
1	17	9102	14090	21040	23200	487900	1	99	1684	41840	19220	2098000	2911	5825
10	19	9232	15070	22660	25800	358800	1	85.5	2020	50240	30120	3564000	1379	3054
11	22	9385	14720	23870	25200	986700	1	79	2371	66470	29700	2033000	1117	3112
12	17	9198	14270	19430	22040	235500	1	64	753	40190	12430	3030000	1801	3729
13	17	8522	13440	18610	21410	414900	1	51.25	1338	57640	25440	2902000	1090	3385
14	18	9618	15350	23780	25960	842900	1	115.5	3389	81160	43240	2724000	751	2559
15	23	9333	14690	21850	25150	461700	1	112.5	2253	57410	24660	1650000	1106	2932
16	17	9017	14850	23360	24980	622500	1	17	83	31490	3040	3484000	1886	2547
17	17	8824	14270	21470	24680	502300	1	69	1285	40970	19270	2507000	1560	2991
18	17	8808	14080	20520	23130	374800	1	46.5	286	21100	6618	3109000	2639	2714
19	22	9392	14640	21420	24400	398300	1	55.5	635.5	97750	44410	3294000	540	2507
2	18	9498	15110	24110	26990	1769000	1	95	1892	49020	24320	3194000	1722	3522
20	17	9258	15100	22760	25870	329000	1	182.2	2377	30470	15120	2191000	1902	2552

21	18	8743	14080	19980	22900	382300	1	47	242	28350	10540	2112000	1745	2541
22	17	9028	13780	19350	22180	280400	1	66	709	93550	32680	4329000	601	3167
23	17	8691	13510	18160	20990	299400	1	81	3000	60270	35770	1846000	864	2876
24	35	9010	14360	20350	23990	299400	1	70	1473	38320	18760	1598000	1241	2339
25	18	8759	13750	20250	21960	811800	1	47	591	37790	12510	1671000	1356	2546
26	17	8301	13450	19560	21400	794900	1	43	658.5	25290	11010	1885000	1538	1990
27	18	9050	13840	19090	22170	393400	1	36	402	28310	13440	1828000	1600	2395
28	59	8450	13480	19260	21880	296600	1	95.5	3234	44550	22760	2540000	914	2135
29	27	9512	14320	19890	23830	174800	1	44.5	548	69690	37260	2187000	599	2098
3	17	9206	13800	20090	23290	331800	1	98.5	2561	69440	30290	3487000	1319	4571
30	17	9027	14330	20740	24060	710100	1	185	3173	41270	24300	1515000	973	1938
31	24	9441	14130	20740	23130	1839000	1	49	560	53160	11380	3197000	749	1909
32	53	9778	15500	23740	27160	386700	1	43.5	451	63920	32820	2068000	603	1631
33	18	9067	13800	19570	22400	495800	1	24	215.5	32850	11310	2705000	954	1602
34	19	9223	13810	17890	21510	272300	1	114	2158	62980	23110	2852000	665	2351
35	17	8573	13390	17490	21360	595100	1	48	373	36500	14600	1562000	697	1511
36	19	9236	14650	21050	24140	285000	1	139.5	644	46020	13710	1646000	644	1462
37	34	8888	13920	20440	23480	485500	1	143.2	2252	55890	24670	2721000	550	1505
38	20	9149	14610	20030	24130	279300	1	38	253.5	25160	5292	3594000	942	1192
4	32	9230	14550	21960	25090	523000	1	107.5	2628	72070	32110	3386000	1219	4014
5	17	9099	14440	20990	24400	347800	1	145	2434	36410	18810	2281000	2360	4228
6	21	9080	14250	21900	24160	762700	1	77	1343	37220	16020	3095000	2063	3525
7	18	9180	14410	20450	23500	599400	1	129	2769	49150	27480	3384000	1647	3953
8	17	9996	16310	24990	27580	976100	1	14	106	38170	9011	2988000	1945	2971
9	39	9546	15400	25340	27270	471200	1	152	1860	26770	14720	1059000	2276	2408

**Labrador Retriever**			cHD							Exome				
Chr	min	Q1	median	mean	Q3	max	min	Q1	median	mean	Q3	max	# SNPs exome	# SNPs cHD
1	17	8984	13810	19810	22440	650000	1	100.2	1682	39780	19170	1954000	3062	6193

10	19	9003	14170	20620	23450	509700	1	88	2224	46740	29550	2067000	1482	3354
11	22	9346	14640	23200	25070	989900	1	72	1970	61110	28170	2214000	1215	3204
12	17	9082	14040	18500	21210	284300	1	58	689	35760	9238	3030000	2024	3916
13	17	8670	13320	17430	20590	277700	1	54.75	1580	56180	28580	2901000	1124	3614
14	20	8906	13840	19020	22010	282300	1	104	1873	62010	32230	2662000	983	3200
15	23	9016	14110	19900	22660	606200	1	114	3217	59160	31610	1650000	1077	3220
16	17	8834	13760	19690	22170	616300	1	17	85	28230	3986	3412000	2104	3022
17	17	8438	13450	18770	21780	285400	1	90	1422	35780	15810	2507000	1786	3422
18	17	8653	13530	19500	21520	475600	1	48	322	21560	6886	2990000	2583	2856
19	22	8916	13390	18250	20800	330900	1	82	1242	88860	34250	3294000	594	2943
2	17	9007	14100	20810	23250	1765000	1	87	1404	39300	17520	3194000	2148	4082
20	31	8905	14400	20430	23680	251500	1	153.8	1818	25020	11270	2191000	2316	2844
21	18	8542	13540	17440	20830	297200	1	49	250.5	24630	8396	2190000	2012	2902
22	17	8870	13400	17410	20260	425400	1	70.5	1255	96040	32130	4329000	638	3520
23	17	8644	13210	16690	19950	267200	1	88	2575	56050	32540	1846000	929	3128
24	32	8470	13300	17310	20740	271500	1	69	1258	32100	15420	1598000	1482	2750
25	18	8714	13550	18120	21220	404900	1	57	958	37170	14410	1882000	1385	2845
26	17	8124	13040	17840	19920	724000	1	36	356	21000	10030	1664000	1852	2182
27	18	8990	14090	18680	22370	221500	1	38	334	24510	9862	1281000	1847	2444
28	18	8273	13190	18350	21480	247600	1	95.25	2910	39790	21060	2542000	1022	2241
29	27	9124	13680	18290	21980	389300	1	54	2141	74470	46770	2102000	561	2281
3	17	8912	13530	18890	21880	353500	1	103	2614	63450	25020	3678000	1438	4862
30	17	8790	13520	18210	22270	253800	1	162.5	2291	34410	21320	1507000	1167	2205
31	19	9360	13740	19230	21510	1839000	1	74.5	1202	51640	11400	3574000	771	2068
32	53	9085	13820	18630	21920	289400	1	52.75	1290	55350	28530	2323000	696	2078
33	18	9004	13630	17560	21100	186900	1	23	213	31690	11770	3072000	989	1786
34	19	8776	13120	16330	19590	272300	1	126.5	2392	59280	23140	2021000	706	2573
35	17	8715	13460	17520	21220	396200	1	55.25	372	36310	13770	1562000	706	1508
36	19	8896	13510	17550	20840	264100	1	130.5	858.5	42270	13670	1683000	710	1754
37	34	8910	13560	18330	21930	281500	1	102	1644	45210	17110	2684000	680	1678

38	29	8892	13470	16950	20360	279300	1	47.75	313	22920	6282	2103000	1040	1409
4	19	8901	13960	18970	22160	205800	1	108	2272	57980	24260	3370000	1521	4650
5	17	8877	13980	19280	22340	257900	1	127	1920	32220	16680	2280000	2667	4600
6	21	9068	13920	20230	22930	626400	1	90	1840	36410	18020	2817000	2115	3816
7	18	8782	13600	18200	21140	531500	1	123.5	2551	44500	24130	3246000	1819	4443
8	17	9336	14630	19760	23750	355500	1	18	140	32440	7720	2903000	2289	3756
9	39	8995	14270	20000	23050	471200	1	143	1657	21780	12110	1841000	2798	3052

Supplementary Table S2. Subsampling of cHD SNPs and effect on r^2 values (chromosome 1)

		LD (r^2)					
# SNPs selected	# SNPs that pass thresholds	min	Q1	median	mean	Q3	max
1500	1115	0	0.04	0.127	0.2458	0.3413	1
3000	2244	0	0.054	0.17	0.3037	0.467	1
4500	3365	0	0.065	0.2	0.3385	0.556	1
6000	4495	0	0.072	0.228	0.3637	0.619	1
7500	5606	0	0.077	0.25	0.3835	0.664	1
9000	6727	0	0.086	0.273	0.4016	0.724	1

Supplementary Table S3. Breed and chromosome-specific LD values (r^2) for exome (exo) and cHD

16 Poodles

Chr	Exo_Q1	Exo_median	Exo_Q3	cHD_Q1	cHD_median	cHD_Q3
1	0.042	0.2	0.758	0.086	0.273	0.724
10	0.04	0.177	0.733	0.072	0.231	0.619
11	0.029	0.147	0.619	0.067	0.235	0.616
12	0.033	0.127	0.559	0.08	0.247	0.636
13	0.04	0.2	0.766	0.093	0.273	0.724
14	0.04	0.1625	0.594	0.075	0.246	0.634
15	0.047	0.197	0.724	0.08	0.2655	0.684
16	0.011	0.14	0.619	0.069	0.2295	0.6
17	0.04	0.168	0.652	0.075	0.238	0.644
18	0.04	0.2	0.802	0.075	0.231	0.6195
19	0.043	0.231	0.771	0.0895	0.249	0.65
2	0.033	0.144	0.5458	0.074	0.238	0.604
20	0.04	0.192	0.7193	0.082	0.247	0.652
21	0.046	0.204	0.7705	0.0765	0.238	0.644
22	0.035	0.162	0.644	0.084	0.289	0.747
23	0.04	0.17	0.686	0.075	0.2295	0.645
24	0.04	0.153	0.602	0.07	0.222	0.559
25	0.037	0.167	0.652	0.074	0.231	0.619
26	0.04	0.2	0.771	0.062	0.2005	0.5342
27	0.04	0.2	0.684	0.0645	0.204	0.535
28	0.04225	0.175	0.6858	0.075	0.228	0.562
29	0.03025	0.149	0.635	0.067	0.2	0.51
3	0.04	0.175	0.684	0.08	0.2425	0.664
30	0.03	0.1375	0.5552	0.067	0.21	0.584
31	0.036	0.138	0.529	0.065	0.191	0.4645
32	0.0405	0.178	0.7565	0.065	0.2	0.532
33	0.035	0.1525	0.5885	0.061	0.196	0.535
34	0.04	0.162	0.6447	0.067	0.209	0.532
35	0.047	0.152	0.664	0.055	0.168	0.4182
36	0.04	0.151	0.594	0.059	0.176	0.4745
37	0.048	0.2	0.747	0.064	0.185	0.529
38	0.03075	0.127	0.5142	0.045	0.144	0.386
4	0.04	0.1655	0.644	0.082	0.258	0.644
5	0.04	0.184	0.771	0.075	0.231	0.616
6	0.04	0.184	0.758	0.078	0.238	0.667
7	0.04	0.179	0.716	0.074	0.238	0.636
8	0.034	0.2	0.716	0.077	0.238	0.664
9	0.043	0.17	0.724	0.075	0.235	0.636

6 Poodles

Chr	Exo_Q1	Exo_median	Exo_Q3	cHD_Q1	cHD_median	cHD_Q3
1	0.065	0.273	1	0.127	0.357	1
10	0.045	0.273	1	0.111	0.295	0.7
11	0.03	0.2	0.7	0.1	0.28	0.7
12	0.045	0.182	0.667	0.111	0.28	0.714
13	0.045	0.273	1	0.127	0.357	1
14	0.048	0.238	0.7	0.1	0.28	0.714
15	0.065	0.273	1	0.124	0.333	1
16	0.008	0.182	0.7	0.107	0.28	0.7
17	0.045	0.238	1	0.107	0.28	0.7
18	0.045	0.273	1	0.1	0.28	0.7
19	0.067	0.28	1	0.1265	0.333	0.714
2	0.04	0.2	0.667	0.1	0.28	0.7
20	0.065	0.273	1	0.111	0.333	0.714
21	0.065	0.28	1	0.111	0.333	0.714
22	0.045	0.259	0.714	0.111	0.357	1
23	0.045	0.238	1	0.111	0.28	0.714
24	0.045	0.25	1	0.1	0.28	0.7
25	0.045	0.238	1	0.1	0.28	0.7
26	0.045	0.273	1	0.096	0.273	0.667
27	0.048	0.273	1	0.1	0.273	0.667
28	0.067	0.273	1	0.111	0.28	0.7
29	0.045	0.238	0.714	0.1	0.273	0.667
3	0.065	0.273	1	0.111	0.333	1
30	0.04	0.2	0.667	0.099	0.273	0.667
31	0.04	0.182	0.6	0.096	0.273	0.6
32	0.065	0.273	1	0.1	0.273	0.667
33	0.03	0.182	0.667	0.096	0.273	0.6
34	0.045	0.238	0.714	0.1	0.28	0.667
35	0.065	0.273	1	0.091	0.238	0.5
36	0.045	0.238	0.714	0.096	0.273	0.667
37	0.065	0.273	1	0.1	0.273	0.667
38	0.045	0.2	0.667	0.067	0.238	0.467
4	0.045	0.2	0.714	0.111	0.333	0.714
5	0.045	0.273	1	0.1	0.28	0.7
6	0.048	0.273	1	0.111	0.295	1
7	0.065	0.273	1	0.1	0.28	0.714
8	0.04	0.273	1	0.111	0.328	0.714
9	0.065	0.273	1	0.111	0.28	0.7

6 Golden

Chr	Exo_Q1	Exo_median	Exo_Q3	cHD_Q1	cHD_median	cHD_Q3
1	0.091	0.357	1	0.127	0.455	1
10	0.065	0.333	1	0.127	0.455	1
11	0.048	0.444	1	0.182	0.455	1
12	0.045	0.28	1	0.127	0.4	1
13	0.111	0.4	1	0.143	0.403	1
14	0.111	0.357	1	0.127	0.467	1
15	0.06275	0.4	1	0.127	0.455	1
16	0	0.25	1	0.167	0.455	1
17	0.107	0.4	1	0.167	0.5	1
18	0.086	0.333	1	0.111	0.357	1
19	0.127	0.4	1	0.182	0.51	1
2	0.096	0.4	1	0.143	0.461	1
20	0.127	0.6	1	0.167	0.467	1
21	0.067	0.357	1	0.127	0.4	1
22	0.065	0.28	1	0.143	0.467	1
23	0.045	0.238	1	0.124	0.333	1
24	0.045	0.28	1	0.115	0.4	1
25	0.091	0.333	1	0.143	0.4	1
26	0.067	0.28	1	0.127	0.357	1
27	0.062	0.333	1	0.1	0.28	0.714
28	0.1	0.4	1	0.127	0.357	1
29	0.065	0.333	1	0.1	0.357	1
3	0.091	0.4	1	0.143	0.455	1
30	0.111	0.4	1	0.127	0.4	1
31	0.065	0.28	1	0.111	0.333	1
32	0.127	0.6	1	0.167	0.5	1
33	0.048	0.273	1	0.127	0.314	0.714
34	0.1	0.333	1	0.127	0.357	1
35	0.074	0.28	1	0.1	0.273	0.6
36	0.1	0.357	1	0.127	0.357	1
37	0.1018	0.357	1	0.127	0.357	1
38	0.067	0.333	1	0.114	0.273	0.7
4	0.127	0.4	1	0.167	0.455	1
5	0.127	0.455	1	0.127	0.455	1
6	0.1	0.455	1	0.143	0.455	1
7	0.1	0.333	1	0.127	0.4	1
8	0.008	0.2	1	0.167	0.467	1
9	0.107	0.455	1	0.127	0.5	1

6 Labrador

Chr	Exo_Q1	Exo_median	Exo_Q3	cHD_Q1	cHD_median	cHD_Q3
1	0.065	0.28	1	0.127	0.4	1
10	0.065	0.28	1	0.127	0.4	1
11	0.045	0.28	1	0.127	0.455	1
12	0.065	0.25	1	0.111	0.357	1
13	0.045	0.273	1	0.115	0.357	1
14	0.045	0.238	1	0.111	0.333	1
15	0.1	0.4	1	0.143	0.4	1
16	0.008	0.183	1	0.127	0.375	1
17	0.08725	0.273	1	0.127	0.357	1
18	0.091	0.333	1	0.125	0.333	1
19	0.1	0.28	1	0.127	0.357	1
2	0.065	0.28	1	0.115	0.367	1
20	0.048	0.28	1	0.111	0.4	1
21	0.045	0.273	1	0.111	0.2845	0.714
22	0.045	0.25	0.714	0.127	0.357	1
23	0.065	0.238	1	0.111	0.28	0.714
24	0.04	0.238	0.7	0.111	0.333	1
25	0.065	0.273	1	0.127	0.4	1
26	0.091	0.28	1	0.1	0.28	0.7
27	0.066	0.28	1	0.1052	0.357	1
28	0.091	0.357	1	0.127	0.357	1
29	0.045	0.182	1	0.111	0.333	0.714
3	0.065	0.28	1	0.127	0.4	1
30	0.067	0.273	1	0.111	0.295	1
31	0.0635	0.273	1	0.096	0.28	0.714
32	0.096	0.4	1	0.115	0.333	0.714
33	0.065	0.28	1	0.096	0.28	0.7
34	0.045	0.238	0.7	0.111	0.28	0.7
35	0.067	0.28	1	0.1	0.28	0.667
36	0.067	0.28	1	0.1	0.28	0.661
37	0.065	0.28	1	0.1	0.333	0.714
38	0.045	0.2	0.7	0.067	0.227	0.5
4	0.048	0.273	1	0.127	0.357	1
5	0.091	0.357	1	0.1248	0.357	1
6	0.091	0.333	1	0.127	0.4	1
7	0.067	0.273	1	0.115	0.333	1
8	0.03	0.259	1	0.115	0.357	1
9	0.067	0.273	1	0.124	0.333	0.714

Supplementary Data S1. WES- and cHD-specific linkage disequilibrium (measured in r^2) relative to position and relation between r^2 and distance for all chromosomes for the Poodle breed (n = 16).

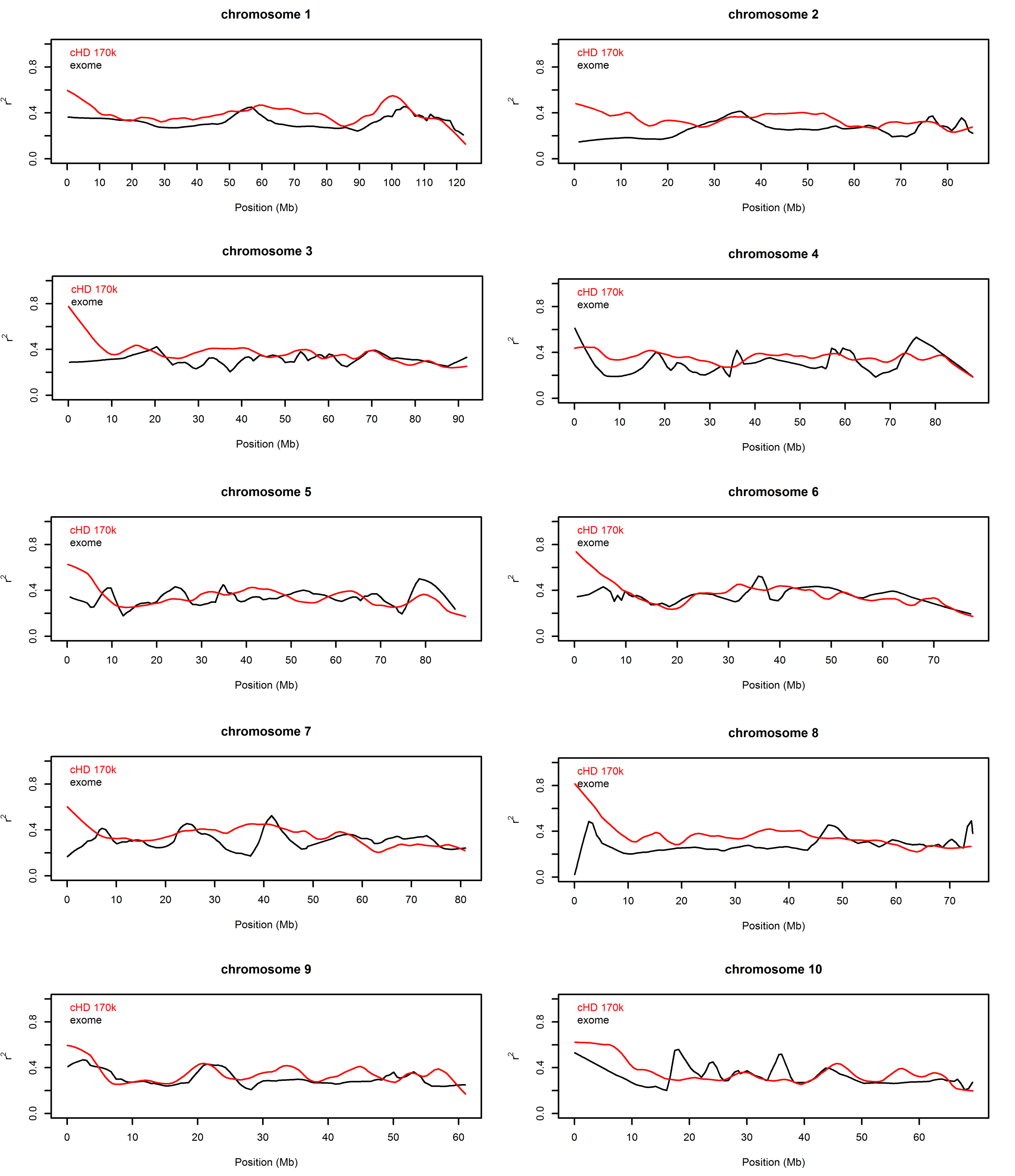
chromosome 1
chromosome 2
chromosome 3
chromosome 4
chromosome 5
chromosome 6
chromosome 7
chromosome 8
chromosome 9
chromosome 10
cHD 170k
exome
r²
Position (Mb)

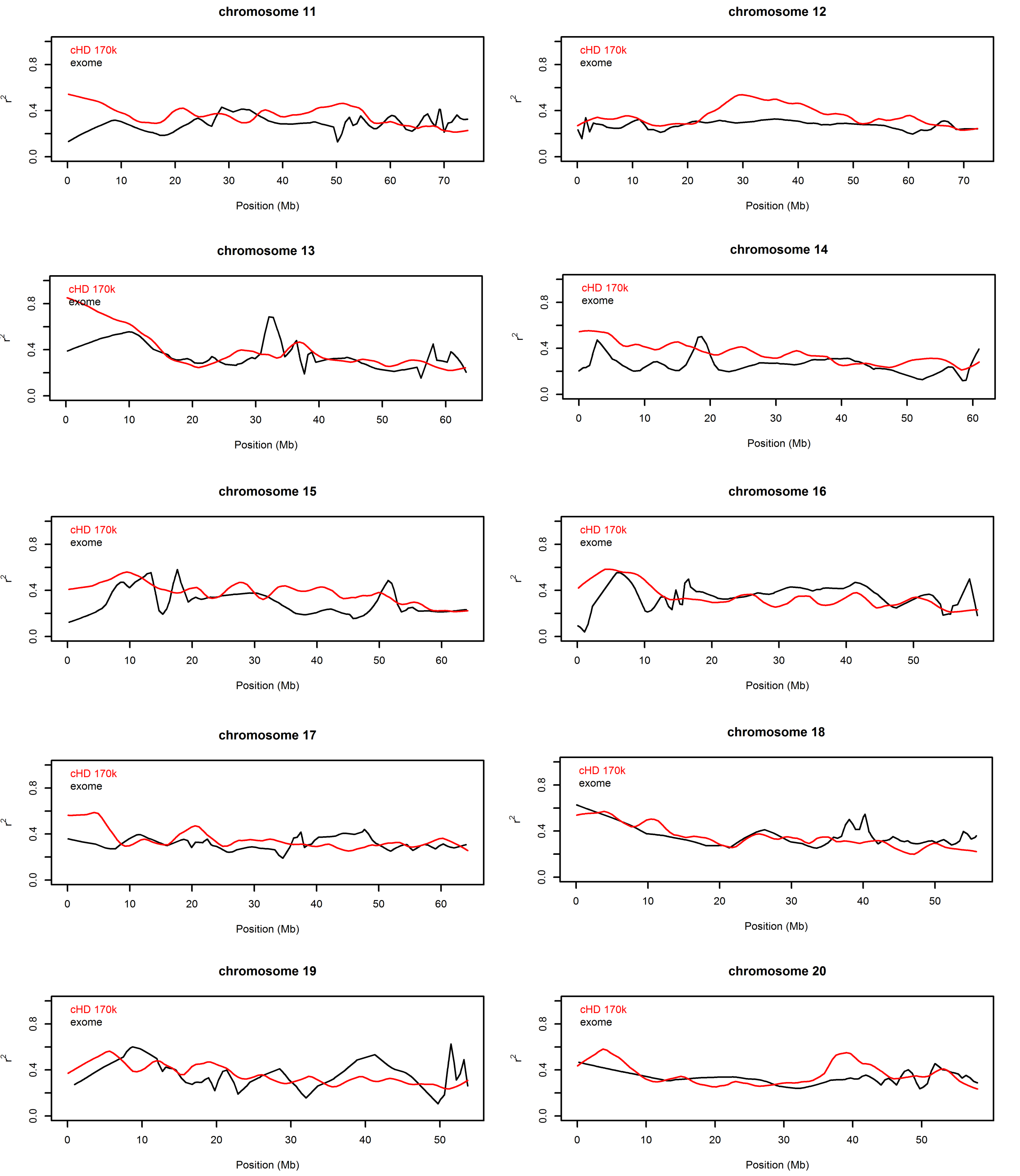
chromosome 11
cHD 170k
exome
r2
0.0 0.4 0.8
0 10 20 30 40 50 60 70
Position (Mb)
chromosome 12
cHD 170k
exome
r2
0.0 0.4 0.8
0 10 20 30 40 50 60 70
Position (Mb)
chromosome 13
cHD 170k
exome
r2
0.0 0.4 0.8
0 10 20 30 40 50 60
Position (Mb)
chromosome 14
cHD 170k
exome
r2
0.0 0.4 0.8
0 10 20 30 40 50 60
Position (Mb)
chromosome 15
cHD 170k
exome
r2
0.0 0.4 0.8
0 10 20 30 40 50 60
Position (Mb)
chromosome 16
cHD 170k
exome
r2
0.0 0.4 0.8
0 10 20 30 40 50
Position (Mb)
chromosome 17
cHD 170k
exome
r2
0.0 0.4 0.8
0 10 20 30 40 50 60
Position (Mb)
chromosome 18
cHD 170k
exome
r2
0.0 0.4 0.8
0 10 20 30 40 50
Position (Mb)
chromosome 19
cHD 170k
exome
r2
0.0 0.4 0.8
0 10 20 30 40 50
Position (Mb)
chromosome 20
cHD 170k
exome
r2
0.0 0.4 0.8
0 10 20 30 40 50
Position (Mb)

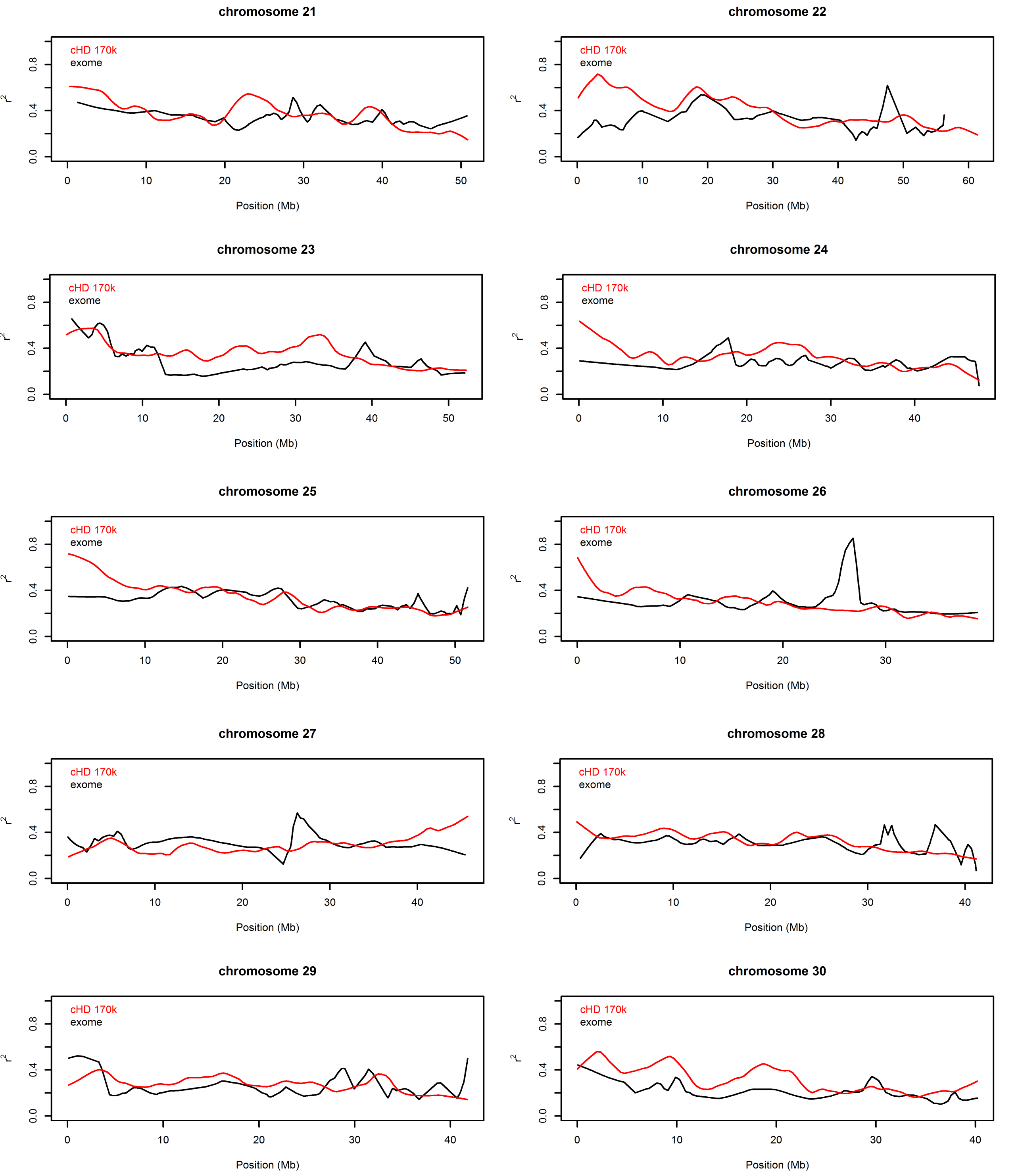
chromosome 21
chromosome 22
chromosome 23
chromosome 24
chromosome 25
chromosome 26
chromosome 27
chromosome 28
chromosome 29
chromosome 30
cHD 170k
exome
r^2
Position (Mb)

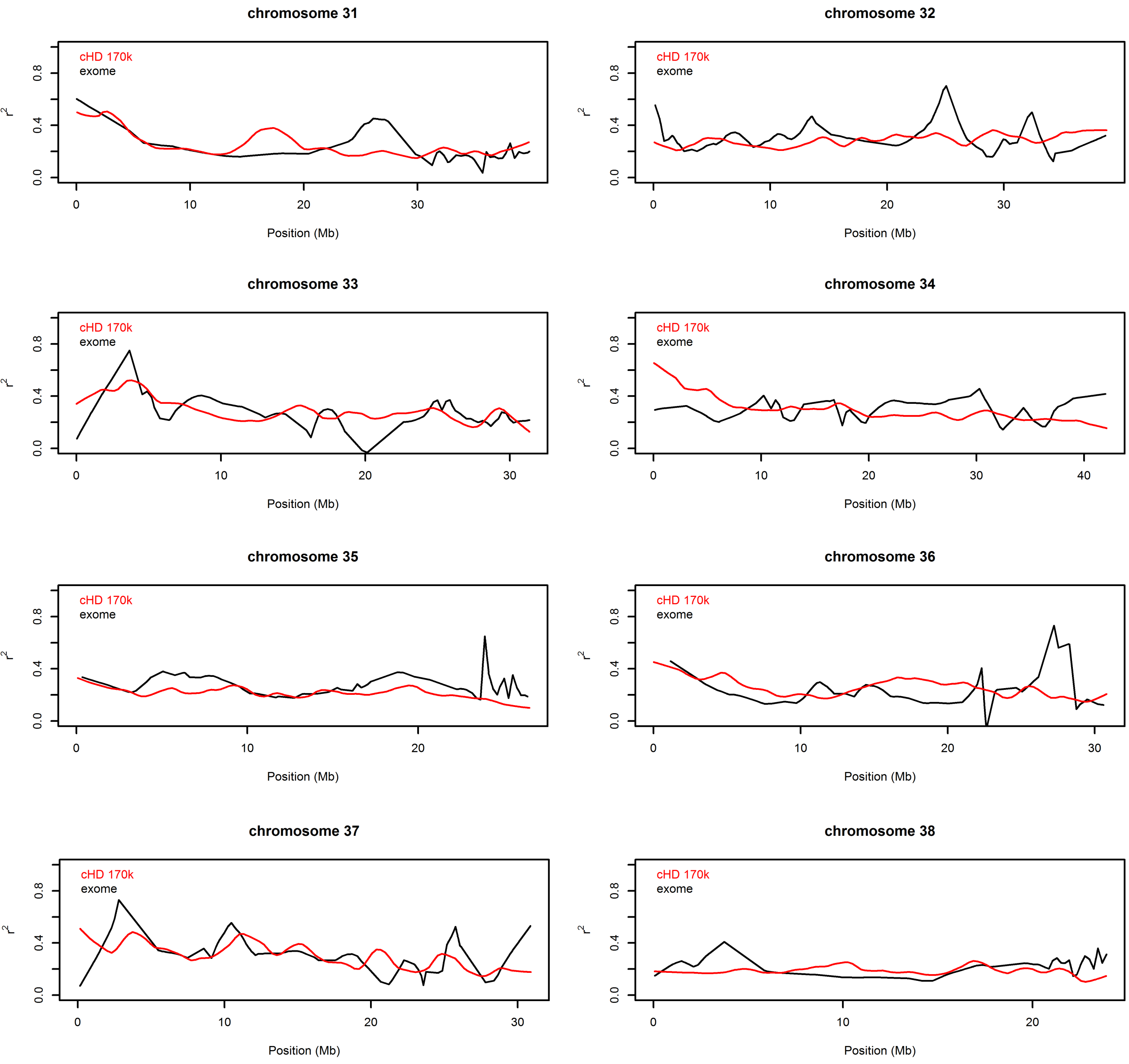
chromosome 31
cHD 170k
exome
r2
0.0
0.4
0.8
0
10
20
30
Position (Mb)
chromosome 32
cHD 170k
exome
r2
0.0
0.4
0.8
0
10
20
30
Position (Mb)
chromosome 33
cHD 170k
exome
r2
0.0
0.4
0.8
0
10
20
30
Position (Mb)
chromosome 34
cHD 170k
exome
r2
0.0
0.4
0.8
0
10
20
30
40
Position (Mb)
chromosome 35
cHD 170k
exome
r2
0.0
0.4
0.8
0
10
20
Position (Mb)
chromosome 36
cHD 170k
exome
r2
0.0
0.4
0.8
0
10
20
30
Position (Mb)
chromosome 37
cHD 170k
exome
r2
0.0
0.4
0.8
0
10
20
30
Position (Mb)
chromosome 38
cHD 170k
exome
r2
0.0
0.4
0.8
0
10
20
Position (Mb)

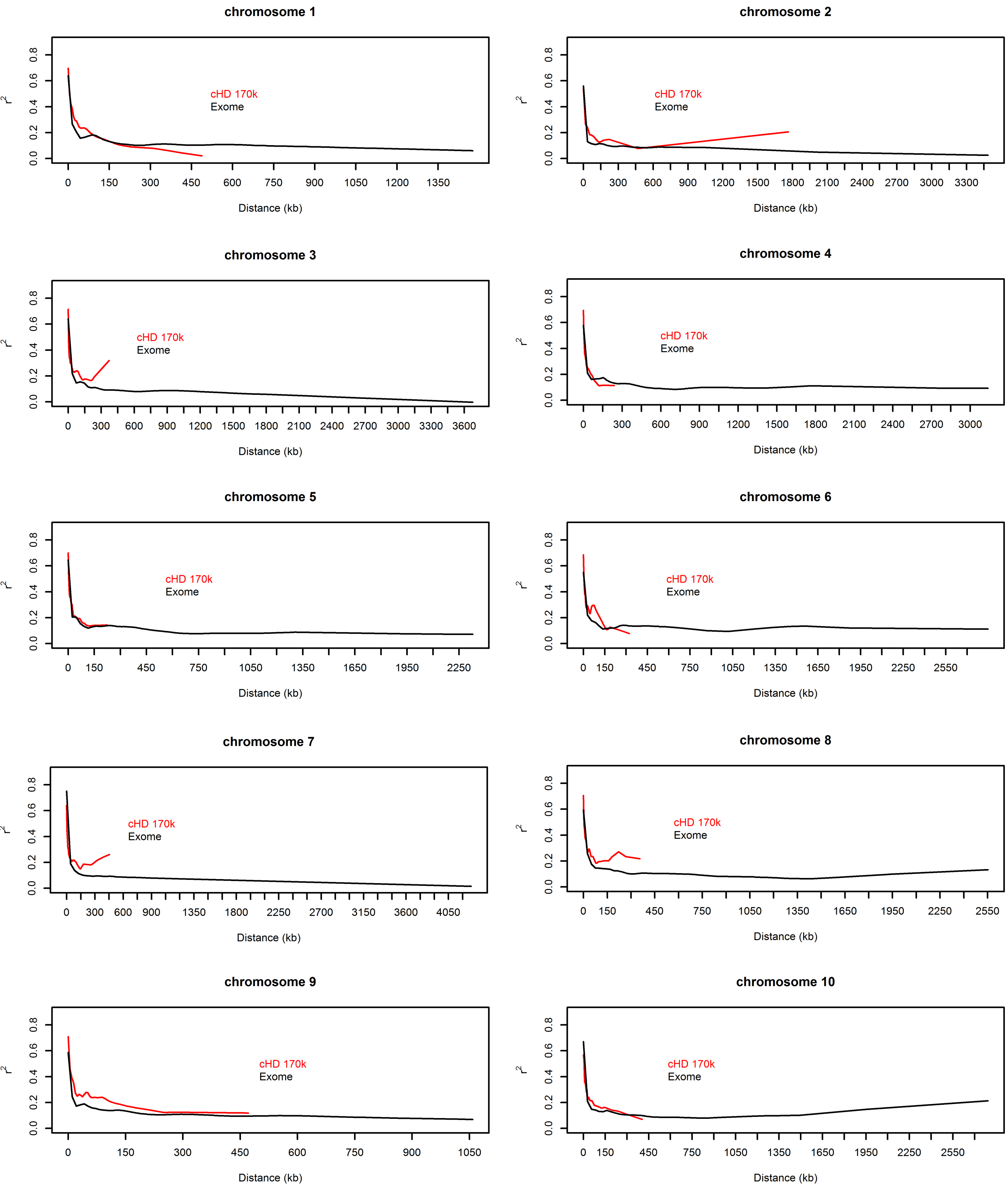

chromosome 1
cHD 170k
Exome
r2
0.0 0.2 0.4 0.6 0.8
0 150 300 450 600 750 900 1050 1200 1350
Distance (kb)
chromosome 2
cHD 170k
Exome
r2
0.0 0.2 0.4 0.6 0.8
0 300 600 900 1200 1500 1800 2100 2400 2700 3000 3300
Distance (kb)
chromosome 3
cHD 170k
Exome
r2
0.0 0.2 0.4 0.6 0.8
0 300 600 900 1200 1500 1800 2100 2400 2700 3000 3300 3600
Distance (kb)
chromosome 4
cHD 170k
Exome
r2
0.0 0.2 0.4 0.6 0.8
0 300 600 900 1200 1500 1800 2100 2400 2700 3000
Distance (kb)
chromosome 5
cHD 170k
Exome
r2
0.0 0.2 0.4 0.6 0.8
0 150 450 750 1050 1350 1650 1950 2250
Distance (kb)
chromosome 6
cHD 170k
Exome
r2
0.0 0.2 0.4 0.6 0.8
0 150 450 750 1050 1350 1650 1950 2250 2550
Distance (kb)
chromosome 7
cHD 170k
Exome
r2
0.0 0.2 0.4 0.6 0.8
0 300 600 900 1350 1800 2250 2700 3150 3600 4050
Distance (kb)
chromosome 8
cHD 170k
Exome
r2
0.0 0.2 0.4 0.6 0.8
0 150 450 750 1050 1350 1650 1950 2250 2550
Distance (kb)
chromosome 9
cHD 170k
Exome
r2
0.0 0.2 0.4 0.6 0.8
0 150 300 450 600 750 900 1050
Distance (kb)
chromosome 10
cHD 170k
Exome
r2
0.0 0.2 0.4 0.6 0.8
0 150 450 750 1050 1350 1650 1950 2250 2550
Distance (kb)

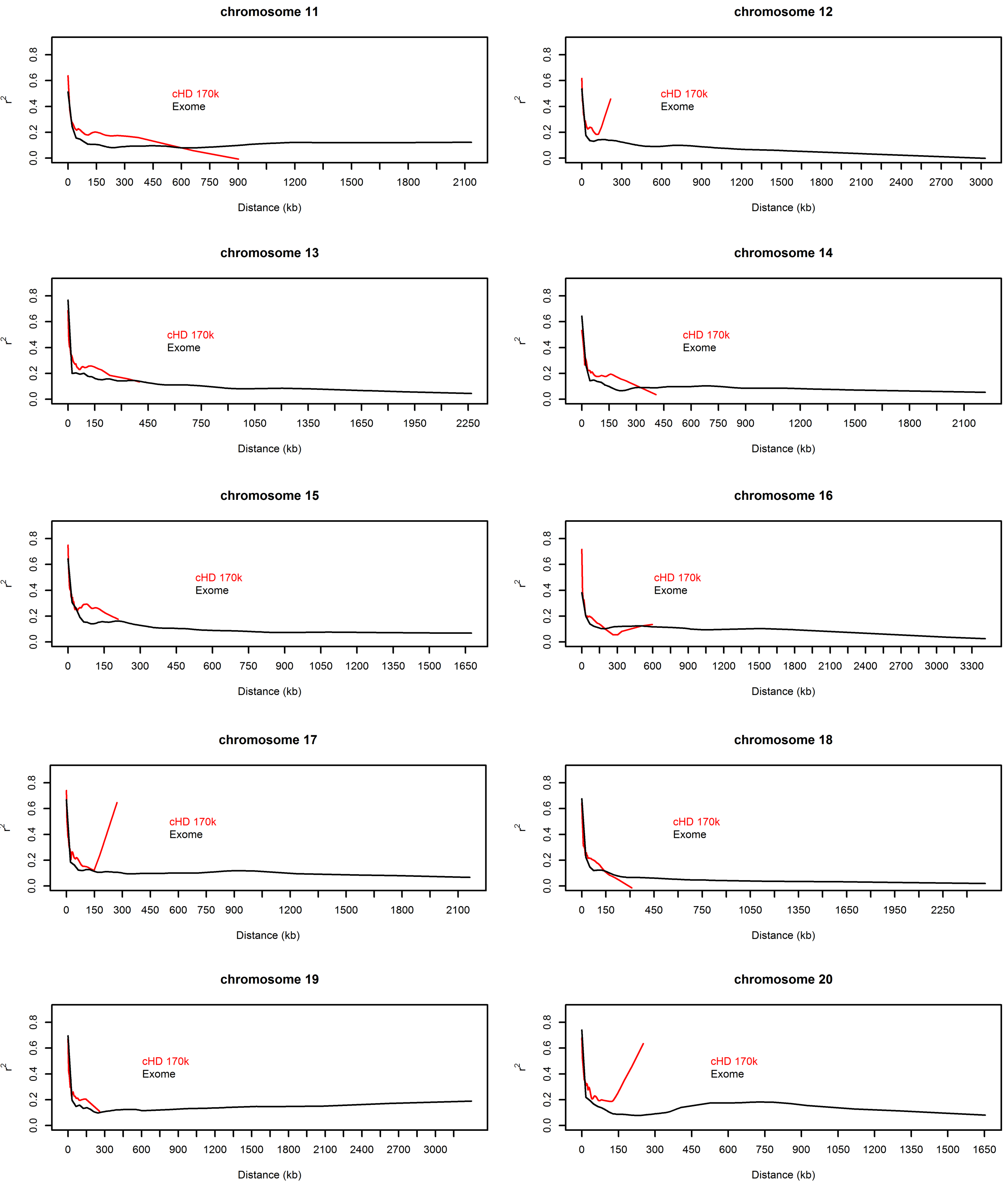
chromosome 11
cHD 170k
Exome
r2
0.0 0.2 0.4 0.6 0.8
0 150 300 450 600 750 900 1200 1500 1800 2100
Distance (kb)
chromosome 12
cHD 170k
Exome
r2
0.0 0.2 0.4 0.6 0.8
0 300 600 900 1200 1500 1800 2100 2400 2700 3000
Distance (kb)
chromosome 13
cHD 170k
Exome
r2
0.0 0.2 0.4 0.6 0.8
0 150 450 750 1050 1350 1650 1950 2250
Distance (kb)
chromosome 14
cHD 170k
Exome
r2
0.0 0.2 0.4 0.6 0.8
0 150 300 450 600 750 900 1200 1500 1800 2100
Distance (kb)
chromosome 15
cHD 170k
Exome
r2
0.0 0.2 0.4 0.6 0.8
0 150 300 450 600 750 900 1050 1200 1350 1500 1650
Distance (kb)
chromosome 16
cHD 170k
Exome
r2
0.0 0.2 0.4 0.6 0.8
0 300 600 900 1200 1500 1800 2100 2400 2700 3000 3300
Distance (kb)
chromosome 17
cHD 170k
Exome
r2
0.0 0.2 0.4 0.6 0.8
0 150 300 450 600 750 900 1200 1500 1800 2100
Distance (kb)
chromosome 18
cHD 170k
Exome
r2
0.0 0.2 0.4 0.6 0.8
0 150 450 750 1050 1350 1650 1950 2250
Distance (kb)
chromosome 19
cHD 170k
Exome
r2
0.0 0.2 0.4 0.6 0.8
0 300 600 900 1200 1500 1800 2100 2400 2700 3000
Distance (kb)
chromosome 20
cHD 170k
Exome
r2
0.0 0.2 0.4 0.6 0.8
0 150 300 450 600 750 900 1050 1200 1350 1500 1650
Distance (kb)

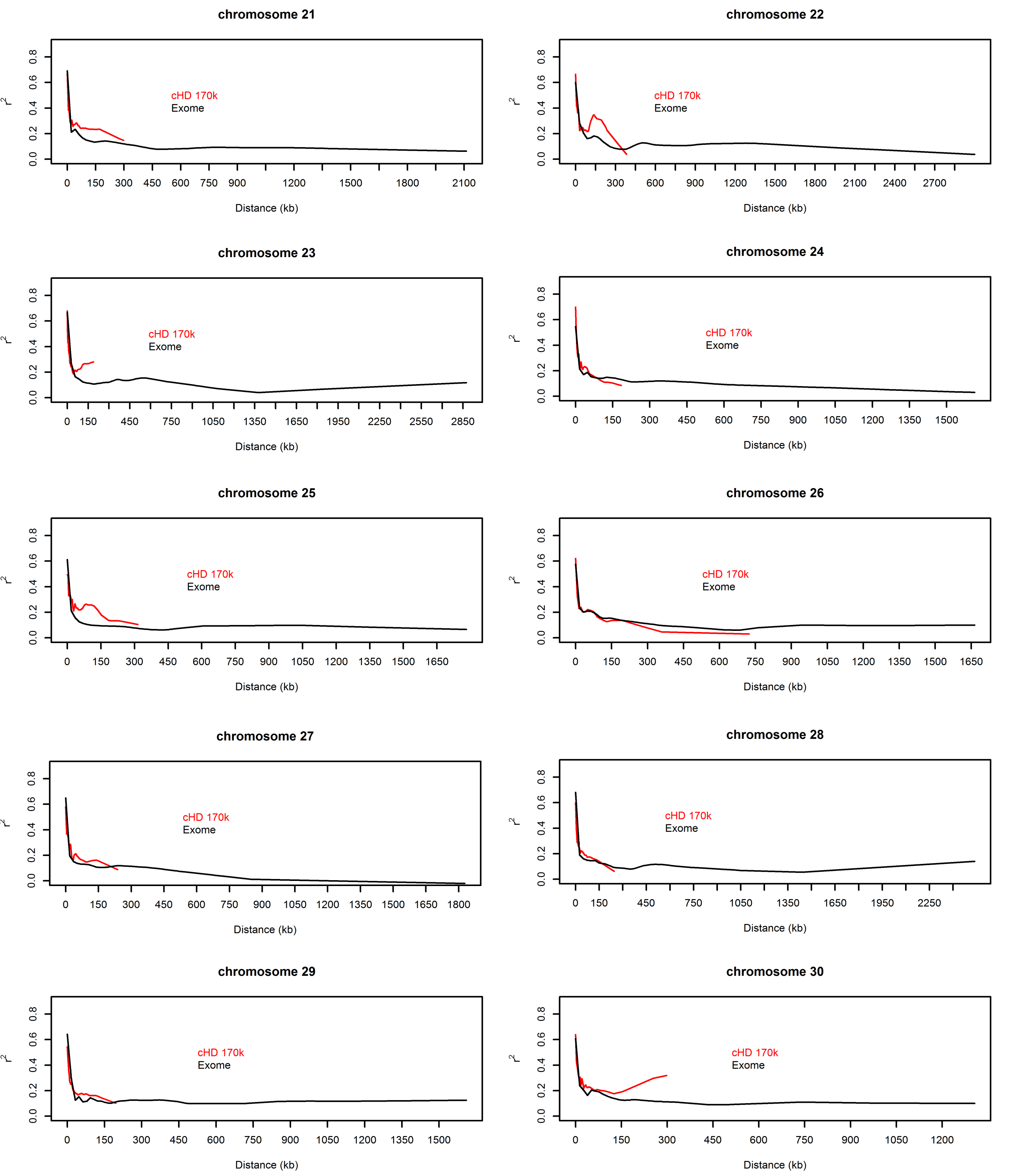

chromosome 21
cHD 170k
Exome
r2
0.0 0.2 0.4 0.6 0.8
0 150 300 450 600 750 900 1200 1500 1800 2100
Distance (kb)
chromosome 22
cHD 170k
Exome
r2
0.0 0.2 0.4 0.6 0.8
0 300 600 900 1200 1500 1800 2100 2400 2700
Distance (kb)
chromosome 23
cHD 170k
Exome
r2
0.0 0.2 0.4 0.6 0.8
0 150 450 750 1050 1350 1650 1950 2250 2550 2850
Distance (kb)
chromosome 24
cHD 170k
Exome
r2
0.0 0.2 0.4 0.6 0.8
0 150 300 450 600 750 900 1050 1200 1350 1500
Distance (kb)
chromosome 25
cHD 170k
Exome
r2
0.0 0.2 0.4 0.6 0.8
0 150 300 450 600 750 900 1050 1200 1350 1500 1650
Distance (kb)
chromosome 26
cHD 170k
Exome
r2
0.0 0.2 0.4 0.6 0.8
0 150 300 450 600 750 900 1050 1200 1350 1500 1650
Distance (kb)
chromosome 27
cHD 170k
Exome
r2
0.0 0.2 0.4 0.6 0.8
0 150 300 450 600 750 900 1050 1200 1350 1500 1650 1800
Distance (kb)
chromosome 28
cHD 170k
Exome
r2
0.0 0.2 0.4 0.6 0.8
0 150 450 750 1050 1350 1650 1950 2250
Distance (kb)
chromosome 29
cHD 170k
Exome
r2
0.0 0.2 0.4 0.6 0.8
0 150 300 450 600 750 900 1050 1200 1350 1500
Distance (kb)
chromosome 30
cHD 170k
Exome
r2
0.0 0.2 0.4 0.6 0.8
0 150 300 450 600 750 900 1050 1200
Distance (kb)

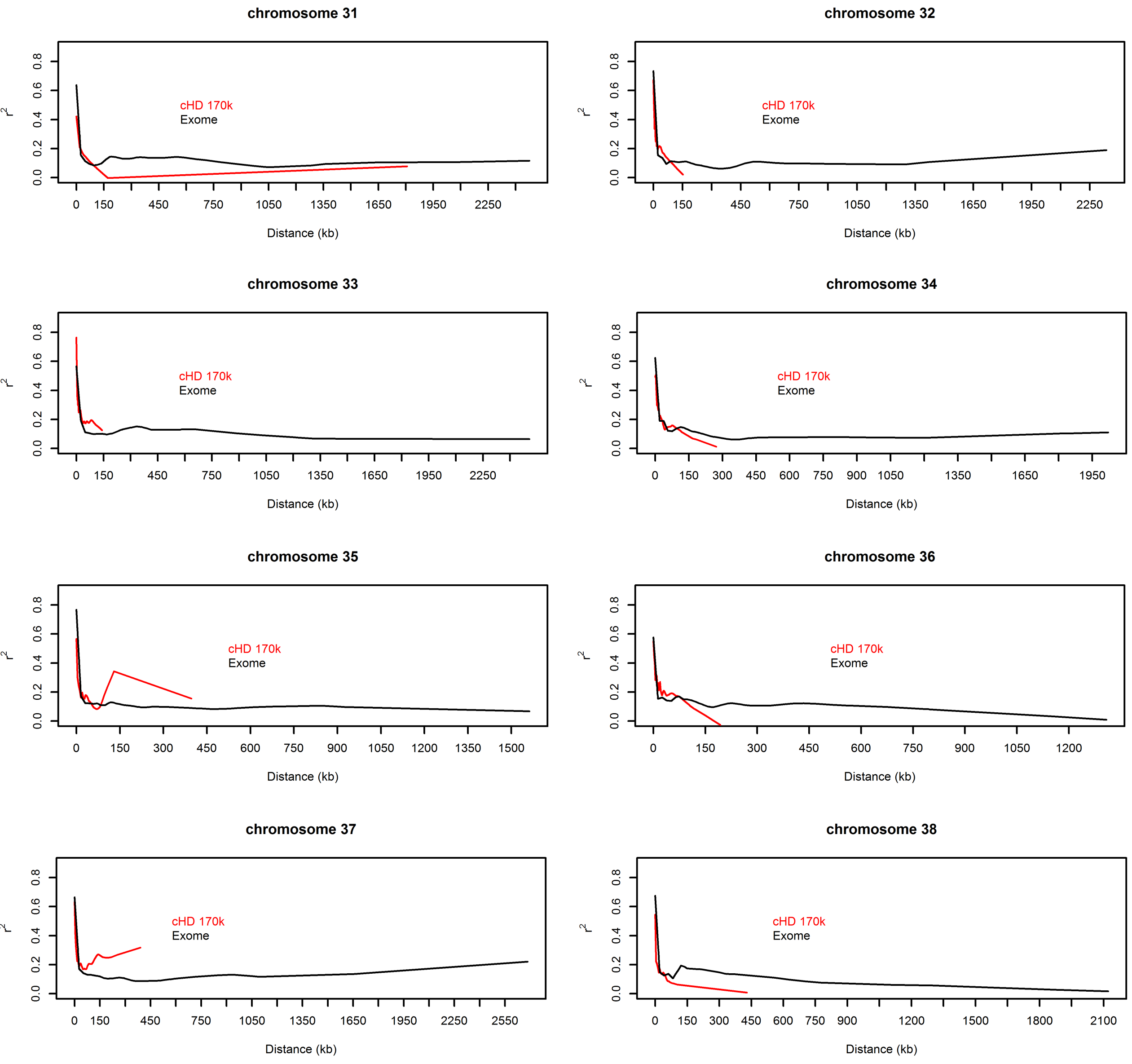

chromosome 31
cHD 170k
Exome
r2
0.0 0.2 0.4 0.6 0.8
0 150 450 750 1050 1350 1650 1950 2250
Distance (kb)
chromosome 32
cHD 170k
Exome
r2
0.0 0.2 0.4 0.6 0.8
0 150 450 750 1050 1350 1650 1950 2250
Distance (kb)
chromosome 33
cHD 170k
Exome
r2
0.0 0.2 0.4 0.6 0.8
0 150 450 750 1050 1350 1650 1950 2250
Distance (kb)
chromosome 34
cHD 170k
Exome
r2
0.0 0.2 0.4 0.6 0.8
0 150 300 450 600 750 900 1050 1350 1650 1950
Distance (kb)
chromosome 35
cHD 170k
Exome
r2
0.0 0.2 0.4 0.6 0.8
0 150 300 450 600 750 900 1050 1200 1350 1500
Distance (kb)
chromosome 36
cHD 170k
Exome
r2
0.0 0.2 0.4 0.6 0.8
0 150 300 450 600 750 900 1050 1200
Distance (kb)
chromosome 37
cHD 170k
Exome
r2
0.0 0.2 0.4 0.6 0.8
0 150 450 750 1050 1350 1650 1950 2250 2550
Distance (kb)
chromosome 38
cHD 170k
Exome
r2
0.0 0.2 0.4 0.6 0.8
0 150 300 450 600 750 900 1200 1500 1800 2100
Distance (kb)

Supplementary Data S2. Choosing a correct window size to detect the causal SNP

Both intuitively and based on the relation between distance and r^2 (Fig. 2(d)), it seems likely that tagSNPs in the neighborhood of the causal SNPs will be more often associated with the causal SNP than distant SNPs. Hence, it can be hypothesized that while distant SNPs do not add to the signal, at some point, if their number becomes large enough, they will mainly reduce significance as they increase the number of tests and, as a consequence, the FWER correction. In addition, as previously demonstrated, using very distant SNPs might even increase the false positive rate[20]. As the signal was simulated in this study, these hypotheses could be characterized in depth.

To evaluate the role of including more distant SNPs, the number of SNPs available to detect the signal was varied and the effect on power was assessed. This was performed by using several window sizes, expressed in number of tagSNPs on each side of the "causal SNP". In total, five different window sizes were used. These window sizes were 10 SNPs on each side of the "causal SNP", followed by 20, 30 and 44 SNPs (corresponding to a total physical distance of ≈ 250 kb, 500 kb, 800 kb and 1 Mb, based on the median distance between cHD SNPs) and the entire chromosome (Suppl. Fig. S1(a)-(d)). The power was evaluated and the distance of the most significant SNP to the causal SNP was recorded.

Our results show that the power remains relatively constant from 1 to 44 SNPs (Suppl. Fig. S1(a, c)), i.e. it does not pay off to use more distant SNPs to detect the signal. When the entire chromosome is used, the power drops markedly and is close to zero. This confirms the previous hypothesis that distant SNPs generally do not increase the signal but they do increase the correction burden. In addition, very distant SNPs might indeed even increase the false positive rate. This statement on false positives is based on the distance between the most significant SNP and the causal SNP. For exonic and non-exonic signals, this distance gradually goes up going from 10-44 SNPs but the maximum distance remains less than 200 kb for the former and less than 500 kb for the latter for both cHD and WES (Suppl. Fig. S1(b)), which corresponds with a distance generally with LD higher than the background LD of 0.043 (Fig. 2(d)). When the entire chromosome is used, the distance went up to 4 Mb (Suppl. Fig. S1(b)), which is

very distant, not only for the Poodle, but even for dog breeds with extreme high values of LD[2], and thus seems to indicate a tendency towards an increase of false positive results[20]. Because we have prior knowledge on the signal location and the experimental results indicate that close SNPs are the ones that generate the signal, the 10 SNP window was used for subsequent power analyses.

Supplementary Data S3. Hardy-Weinberg testing procedures: to flag or to remove?

Testing for departures of Hardy-Weinberg equilibrium (HWE) is part of the standard procedures when a GWAS is performed. As part of the quality control, the aim is to identify genotyping errors, i.e. departures from HWE are considered to be indicative for genotyping errors[52]. In this section, we comment extensively on the rationale of this test and our choice to not remove SNPs that deviate from HWE, but to flag them.

Firstly, we focus on potential reasons for deviations from HWE. Deviations from HWE can result from genotyping error, true associations (which is what one wants to detect), population stratification and finally, by chance (because of doing a lot of tests)[48,52]. As such, a deviation from HWE cannot be solely attributed to a genotyping error. More mathematically, a SNP for which HWE is tested and HWE is rejected ≠ genotyping error or P(genotyping error| HWE rejected) ≠ 1. Also the converse is not true: removing all SNPs that deviate from HWE does by no means guarantee that all genotyping errors have been removed as genotyping errors can perfectly occur under HWE[49]. Mathematically, a SNP for which HWE is tested and HWE is not rejected ≠ no genotyping error or P(no genotyping error| HWE) ≠ 1. Furthermore, as mentioned earlier, a significant deviation from HWE can actually reflect a true association. This is actually demonstrated by the fact that here the causal variant for hair length, discovered with EG-GWAS, deviated significantly from HWE. Combining these arguments, we conclude that just simply removing SNPs that deviate significantly from HWE is not ideal and not recommended, something which has been stated already several times[50-52].

There are however additional reasons to perform HWE testing with caution. For EG-GWAS, it is important to consider the nature of the data when talking about discovering genotyping errors. EG-GWAS is based on variants discovered during sequencing. For sequencing data, there are far better options to remove errors than HWE deviations, as pointed out in the GATK "Best practices". Instead of HWE deviations, the filtering options can take for example sequencing depth, variant calling quality values, mapping quality, strand bias and quality by depth into account. HWE deviations are thus not part of the analysis pipeline for WES data to find sequencing errors.

The next points consider the potential bias that might occur by either performing or not performing HWE testing. In case we choose the latter option, are our results biased when we decide to keep SNPs that deviate from HWE? For that case, Fardo et al. demonstrated that "… that random miscalls of null SNPs, independent of the phenotype, do not induce bias in case–control or cohort studies, and we suggest that a significant HWE test should not prevent a SNP from being tested when conducting genome-wide association studies in these scenarios"[51].

Next, we consider the other option: are the results biased when we do remove SNPs that deviate from HWE? The answer is yes, and this stresses again the risk of plain removal of SNPs instead of flagging: true associations can also result in HWE deviations. Indeed, in this study, the causal variant for hair length that was high quality sequenced and passed all QC that is normal for sequencing and that was directly discovered with EG-GWAS, deviates significantly from HWE. Just simply removing that SNP would thus bias our results: we would have removed the causal SNP, a true direct association, a deviation from HWE due to association.

Altogether, we conclude that there are no reasons to just blindly remove all SNPs that deviate from HWE. Finally, aside from all these other points, we would like to point out that the power to detect genotyping errors with HWE is generally low, even when sample sizes are extremely large[48].
